# On-site sensing for aflatoxicosis poisoning via ultraviolet excitable aptasensor based on fluorinated ethylene propylene strip: a promising forensic tool

**DOI:** 10.1038/s41598-024-68264-3

**Published:** 2024-07-29

**Authors:** Nur-Fadhilah Mazlan, Edison Eukun Sage, Nur Syamimi Mohamad, Mukram Mohamed Mackeen, Ling Ling Tan

**Affiliations:** 1 Southeast Asia Disaster Prevention Research Initiative (SEADPRI), Institute for Environment and Development (LESTARI), Universiti Kebangsaan Malaysia, 43600 Bangi, Selangor Malaysia; 2 Department of Applied Physics, Faculty of Science and Technology, Universiti Kebangsaan Malaysia, 43600 Bangi, Selangor Malaysia; 3 Department of Chemical Sciences, Faculty of Science and Technology, Universiti Kebangsaan Malaysia, 43600 Bangi, Selangor Malaysia; 4 Institute of Systems Biology, Universiti Kebangsaan Malaysia, 43600 Bangi, Selangor Malaysia

**Keywords:** Aflatoxicosis, Fluorinated ethylene propylene, Forensic, In silico, On-site monitoring, UV-excitable, Chemistry, Materials science

## Abstract

The environmental contamination by extremophile *Aspergillus* species, i.e., Aflatoxin B1, is hardly controllable in Southeast Asia and Sub-Saharan Africa, which lack handling resources and controlled storage facilities. Acute aflatoxicosis poisoning from aflatoxin-prone dietary staples could cause acute hepatic necrosis, acute liver failure, and death. Here, as the cheaper, more straightforward, and facile on-site diagnostic kit is needed, we report an ultraviolet-excitable optical aptasensor based on a fluorinated ethylene propylene film strip. Molecular dynamics on the aptamer.AFB1 complex revealed that the AFB1 to the aptamer increases the overall structural stability, suggesting that the aptamer design is suitable for the intended application. Under various influencing factors, the proposed label-free strategy offers a fast 20-min on-site fabrication simplicity and 19-day shelf-life. The one-pot incubation provides an alternative to catalytic detection and exhibited 4 times reusability. The recovery of crude brown sugar, processed peanuts, and long-grain rice were 102.74 ± 0.41 (n = 3), 86.90 ± 3.38 (n = 3), and 98.50 ± 0.42 (n = 3), comparable to High-Performance Liquid Chromatography-Photodiode Array Detector results. This study is novel owing to the peculiar UV-active spectrum fingerprint and the convenient use of hydrophobic film strips that could promote breakthrough innovations and new frontiers for on-site/forensic detection of environmental pollutants.

## Introduction

Toxic environmental contaminants associated with rapid urbanization, agriculture activities, and heavy industrialization posed concerning adverse health effects worldwide^1^ such as dyes^2–5^, heavy metal ions^6–11^, spilled oil^12–14^, and fungal toxins^15,16^. Owing to high water solubility and the presence of other complex pollutants heavily loaded in the water bodies, potentially cancerous dyes from cosmetics, food, textiles, and paper often underwent polymer composite-based treatment to be converted into harmless products before disposal^2–5^. Heavy metals are non-biodegradable, cannot be broken down, and possess a lengthy half-life where adsorbents such as metal hydroxides, metal oxides, and polymer composites have been incorporated in the elimination strategy to hinder prolonged exposure or bioaccumulation^6–11^. There have been petroleum-degrading microorganisms, as well as superhydrophobic siloxane-inspired composite purification efforts of spilled petroleum-derived oil, a complex mixture of thousands of chemical components including potentially cancer-causing volatile organic compounds and major persistent-occurring polycyclic aromatic hydrocarbons organic pollutants^12–14^.

Aflatoxin B1 (AFB1) is a naturally occurring fungal metabolite produced by extremophilic *Aspergillus* species that tends to thrive in regions with high humidity and temperature environmental conditions. Consequently, subtropical and tropical countries such as Southeast Asia and Sub-Saharan Africa often face higher levels of contamination, which lack the required resources and controlled storage facilities to preserve food in temperature-controlled and dry environments. In view as an agricultural contaminant, the chronic (long-term) aflatoxicosis poisoning exhibited immunological and nutritional effects. In contrast, exposure to excessive amounts of aflatoxin-prone dietary staples is traditionally linked to acute hepatic necrosis, acute liver failure, and even death. Both chronic and acute doses can contribute to an increased cumulative cancer risk^15,16^.

Oxidized AFB1 is capable of permanently damaging the nucleotides by forming an adduct at G G-rich sequence, which led to the development of many detection techniques in many food and dairy products, including rice^17^, dried fruits, peanuts, Figs^18^, oil^19^, spices^20^, and corn^21^. The well-established conventional approaches such as Liquid Chromatography coupled to Mass Spectrometry (LCMS)^22,23^, High-Performance Liquid Chromatography-Fluorescence Detection (HPLC-FD), and Enzyme-Linked Immunosorbent Assay (ELISA)^24^ faced limitations, including laborious, time-consuming, require skilled human resources, running cost, machine complexities, and non-accessible^25^. Thus, an inexpensive, more straightforward, and efficiently operated alternative diagnostic version to detect environmental pollutants is more desirable for on-site or forensic detection.

For on-site AFB1 sensing, some of the recent developments were based on colorimetric^26^, lateral flow strip^27^, polyaniline (PAni) support matrix-based impedimetric sensor^28^, quantum dot nanobead-based fluorescent strip immunosensor^29^, and conducting polymer composite electrode^25^. A genosensor developed for on-site AFB1 sensing in grains utilized DNA-functionalized cryogel for colorimetric detection^26^. The construction of an aptamer-based lateral flow strip for the detection of AFB1 in corn and wheat was based on the competition between AFB1 and fluorescein-labeled complementary DNA strands for affinity binding^27^. In addition, a PAni support matrix-based impedimetric aptasensor, fabricated via surface modification of a screen-printed carbon electrode (SPE), has been documented for the detection of AFB1 in pistachio nuts, cinnamons, cloves, corn, and soybeans^28^. For AFB1 sensing in lotus seeds via the quantum dot nanobead (QBs)-based fluorescent strip immunosensor, it was constructed by employing carboxylated QBs as the fluorescent markers, also bovine serum albumin-AFB1 antigens and goat anti-mouse IgG antibodies were coated on the nitrocellulose (NC) membrane to prepare the test and control lines, respectively^29^. A conducting polymer composite electrode, poly(3,4-ethylenedioxythiophene):polystyrene sulphonic acid, was used for electrochemical AFB1 detection in button mushrooms and okra^25^.

Biotechnology and nanotechnology are the two most promising technologies, and their fusion brings about a novel nanobiotechnology platform, offering opportunities to employ polymer nanocomposites with unique properties tailored for on-site sensing of environmental contaminants^29–33^. One of the recent polymer nanocomposite examples, the conductive networks of polyethylene-multi-walled carbon nanotubes were employed in a vapor sensor, to identify organic solvent vapors of acetone and xylene via ultrasonication anchoring and compression molding techniques^34^. A fluorescent sensor to detect Fe^3+^ ions was developed under UV irradiation, by which carboxyl-functionalized poly(arylene ether nitrile)-based lanthanide coordination polymer nanofibrous membrane was prepared through electrospinning method^35^. Another example is the exploitation strategy of a series Zn(II)-coordination polymers for trace detection of Pd^2+^ by means of fluorescence quenching in aqueous medium^36^. Aptamers have shown notable merits of non-immunogenicity, ease of synthesis, resistance to degradation and denaturation, and antibody-mimicking functional characteristics^37–42^. In a label-free electrochemical aptasensor for Pb^2+^ sensing, the index signal of Pb^2+^ reduced peak was generated by the G-quadruplex conformational change phenomenon, upon stepwise assembly of titanium carbide, gold nanoparticles, and thiol-modified aptamer of Pb^2+10^. The notable synergistic effect of silver plasmon resonance and p–n heterojunction were utilized in the construction of photoelectrochemical aptasensor for detecting chloramphenicol, by strategy of silver-bismuth oxyiodide-titanium dioxide nanorod array as the visible light active material and C = N structure as the recognition unit of chloramphenicol aptamer^43^.

Meanwhile, it is also interesting to note the latest scientific findings implementing polymer nanocomposites in the biomedical/medical field^44–46^, also for detecting inorganic^47,48^ or organic compounds^49,50^. The microwave-based non-equilibrium heating method rendered an ultrafast electrochemical biosensing of COVID-19 infection, allowing SARS-CoV-2 gene detection on zinc sulfide-graphene nanocomposite^44^. Electrocardiogram of high-quality signals can be acquired continuously within 24 h by metalizing the surface of poly(vinyl alcohol) hydrogel with silver nanowires upon fabrication of semidry electrodes under very low impedance^45^. A metamaterial-based sensor fabricated with copper combination square ring microstructures, prepared using lithography and magnetron sputtering approaches, enabled terahertz wave bio-detection of biomolecules with varying refractive indices^46^. Fluorescence quenching strategy of nitrogen-doped carbon dots resulted from the inner filter effect, between permanganate inorganic anion and nitrogen-doped carbon dots, led to the recoveries of 99.42 to 101.16% inorganic anion in contaminated water^47^. The tetrametallic PtNiCuCo alloy nanoparticles that were spray deposited on fluorine-doped tin oxide substrates showed excellent electro-oxidation activity, for catalysis-based sensing of inorganic hydrogen peroxide^48^. Platinum nanoparticle-modified molybdenum disulfide-silica nanocomposites possess excellent peroxidase-like activity, in which the colorimetric identification of organic hydroquinone via discolored product of bluish oxidized 3,3′,5,5′-tetramethylbenzidine was made possible^49^. An electrochemical platform for sensing tetrabromobisphenol A selectively in water samples was devised, through dropping of molecularly imprinted polymer onto MXene and gold nanoparticle modified glassy carbon electrode, giving recovery values of 97.1–106% of this organic pollutant^50^.

Chitosan (CT, 1526.464 g mol^−1^, C_56_H_103_N_9_O_39_) possesses abundant free amine (NH_2_) and hydroxyl (OH) groups that provide good characteristics such as hydrophilicity, film-forming, mechanical strength, antibacterial, biocompatibility, and biodegradability^51–54^. For instance, high mechanical antibacterial hydrogels constructed from chitosan/poly(acrylamide-[2-(methacryloyloxy)ethyl]trimethylammonium chloride), exhibited excellent efficacy against Gram-negative (*Escherichia coli*) and Gram-positive (*Listeria monocytogenes*) bacteria^55^. Cellulose acetate-chitosan bio-composite porous nanofibers were used as adsorbent materials for chromium ions, utilizing synergistic action of the electrostatic forces, hydrogen bonding, and interfacial compatibility of both nanofiber counterparts^56^. The superparamagnetic properties of chitosan-coated cobalt/zinc/ferrite nanoferrofluid composites triggered apoptosis and cancer cell death, in which chitosan moieties assisted entry to the cell^57^. Solid state chitosan-based carbon dots inherited resistance to aggregation-caused quenching, advantageous for white light-emitting diode, attributed by the basic backbone structure of chitosan components, that prevented the π–π interaction between carbon cores^58^. Graphene oxide (GO) is a negatively charged adsorbent sheet of layered nanomaterials with a high surface area-to-volume ratio. GO alone is often hardly collectible from water owing to the abundance of polar functional groups, which give a good dispersion stability and hydrophilic nature to the sheets^59^. CT can interact electrostatically with GO sheets and enhance ease of collection from the water, allowing deposition of graphene oxide cross-linked chitosan (GO-CT) nanocomposite for aptasensor fabrication^51–54^.

Nowadays, the technological frontier of predictive analytics is incorporated virtually by industries and is also reliable in diverse fields, including automotive, aerospace, agriculture, stock market, finance-oriented, retailing, hospitality, medical research, neuroscience, text mining, and weather forecasting^60^. The likelihood of an earthquake with magnitudes of 6 or higher was predicted using the machine-learning algorithm models, such as the k-nearest neighbors, support vector machines, random forests, and XGBoost algorithm^61^. An electromagnetic simulation-numerical optimization strategy was proposed to forecast multilayer radar-absorbing materials with strong absorption, lightweight, and wide absorption bandwidth^62^. A 3-dimensional micropattern-guided transition from surface wrinkle to post-wrinkling bifurcation on elastic bilayer towards a more tunable morphological transition revealed some subcritical patterns for generating creases^63^. In addition, molecular dynamics at the micro-level simulation was utilized to design a new melt-cast explosive with low sensitivity and high energy attributes, using 3,5-difluoro-2,4,6-trinitroanisole as the casting carrier and 2,4,6,8,10,12-hexanitrohexaazaisowurtzitane as the high-energy component^64^. A two-dimensional computational fluid dynamics transient vacuum membrane distillation model was developed, to theoretically guarantee an operation of high thermal efficiency and enhanced membrane distillation flux^65^. The curing-induced residual stresses of the nanoparticle, interphase component, and matrix on the mechanical effect of polymer nanocomposites were studied on a theoretical basis via an in-situ curing process using a molecular dynamics approach^66^.

**Figure 1:**
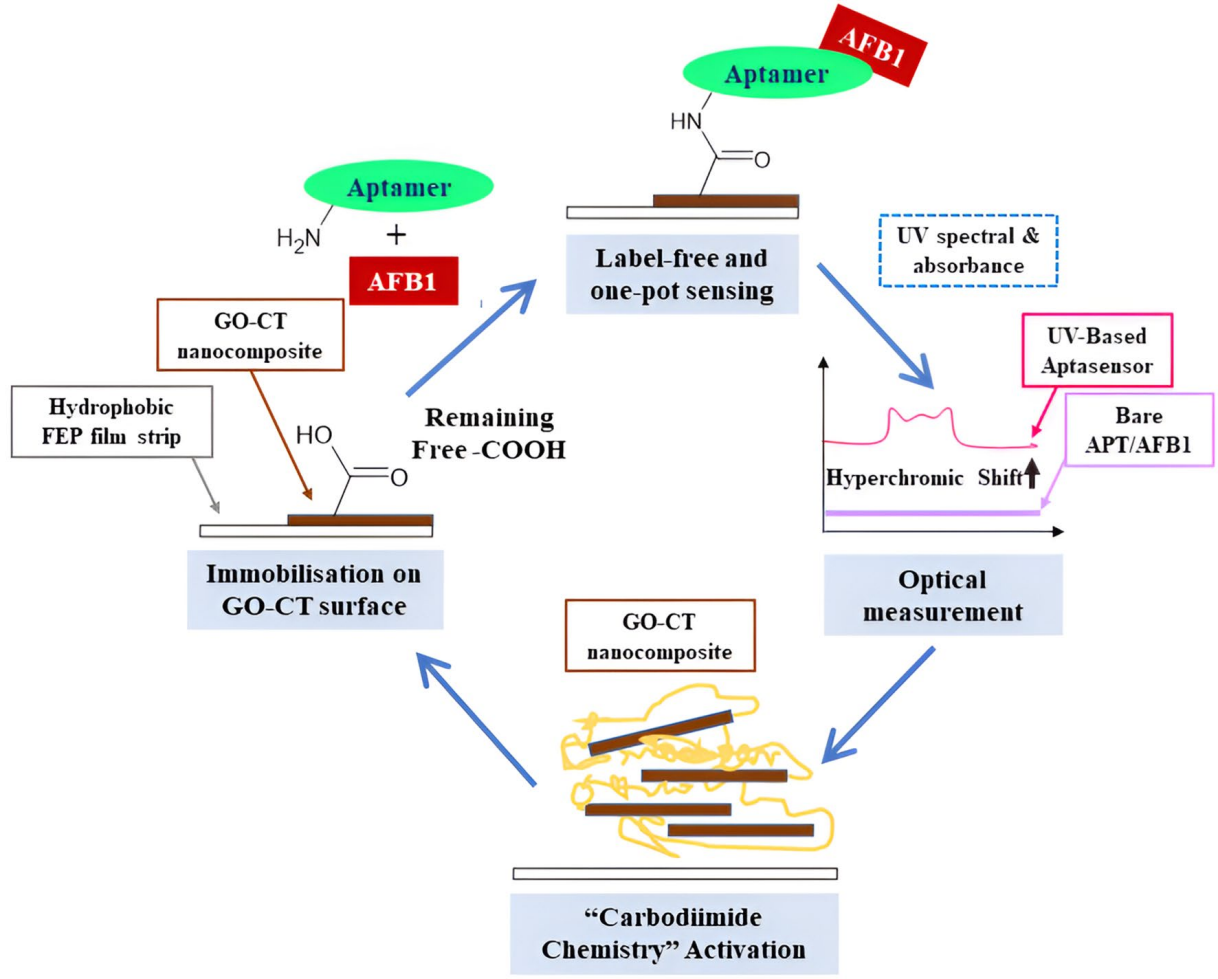
Schematic illustration of UV-based aptasensor in sensing target AFB1 by employing FEP film strip. The aptasensor with conducting GO-CT undergoes electronic excitation when exposed to UV light during measurement. In contrast, no excitation signal when exposed to visible light.

The lightweight, transparent, chemically inert film of fluorinated ethylene propylene (FEP) is a copolymer of tetrafluoroethylene (CF_2_=CF_2_) and hexafluoropropylene (CF_2_ = CF–CF_3_). FEP has been used in aerospace, solar thermal collectors, neutrino detectors, and biomedical applications. It possesses excellent optical and electrical properties, exhibits low friction and anti-stiction properties, and can withstand extreme temperature conditions^67–70^. For instance, it has been incorporated in a triboelectric nanogenerator^67^, neutrino detector^68^, and as a microfluidic strip for IgG antibody detection^69^. Herein, we have performed molecular docking and dynamic analyses of AFB1-aptamer to predict and visualize our sensing mechanism. Docking analyses were performed to investigate the possible interaction of the ligand AFB1 with its receptor, i.e., aptamer, followed by molecular dynamic simulation to assess the aptamer-AFB1 complex stability. This study is novel as we are the first to report ultraviolet (UV)-excitable aptasensor based on fluorinated ethylene propylene strip for on-site/forensic application sensing of aflatoxicosis poisoning. The film strip efficiently probes the solid-state fabricated surface of GO-CT nanolayer by employing N-(3-dimethylaminopropyl)-N′-ethyl-carbodiimide-hydrochloride (EDC) / N-hydroxysuccinimide (NHS)-mediated coupling reactions conjugated to GO-CT nanocomposite surface as illustrated in Fig. 1. Owing to the hydrophobic nature of FEP and hydrophilic nature of GO-CT, fabrication basis of GO-CT by loading technique at a perpendicular angle (90°) was needed to ensure a uniform distribution of GO-CT over the FEP surface, as well as to avoid spillage upon drying, prior to one-pot carbodiimide assisted activation between capturing probe of 50-mer NH_2_-modified aptamer immobilized on GO-CT and targeted pollutant.

## Results and discussion

### Docking theoretical investigations of 50-mer aptamer specificity containing key binding bases for AFB1 molecule

**Figure 2:**
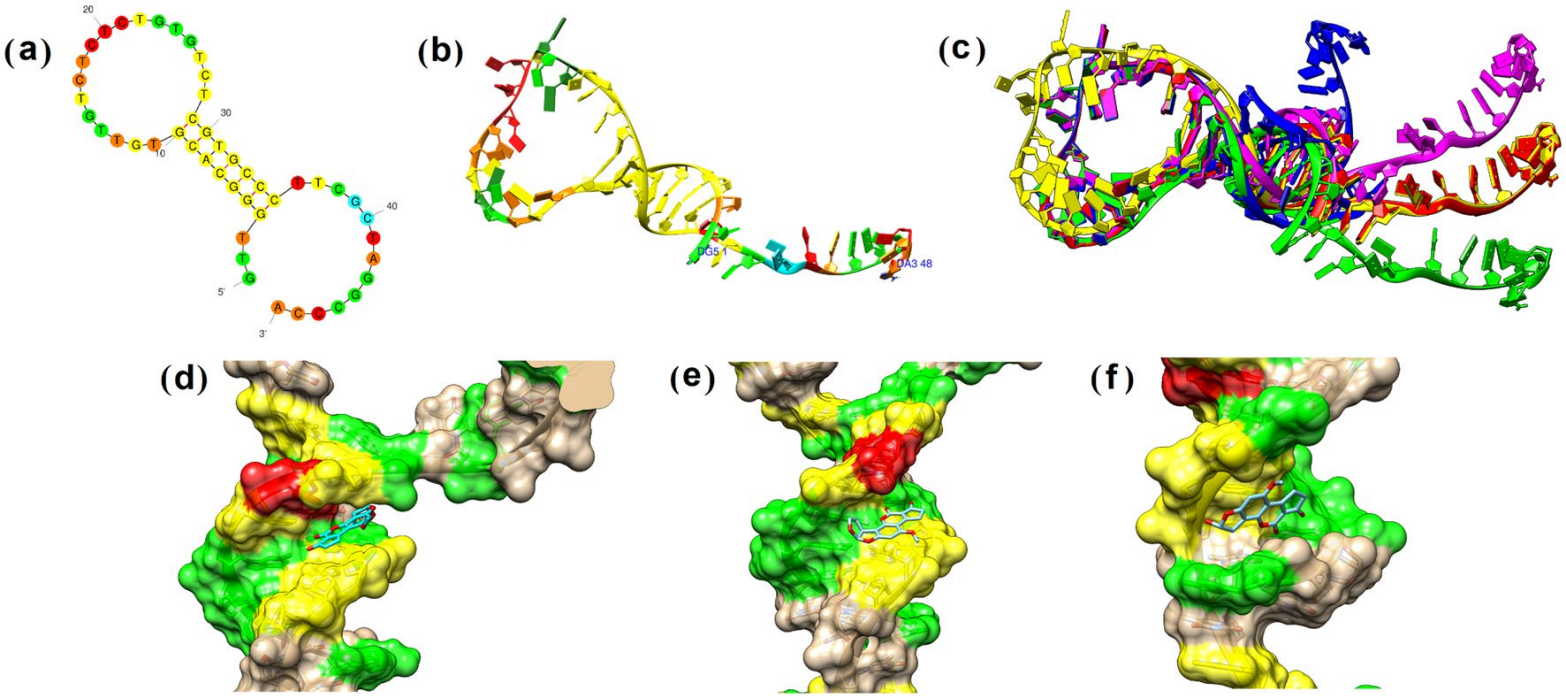
Aptamer structure modelling and molecular docking results. (**a**) Mfold model. (**b**) 3D structure from 3dRNA v2.0 web server. (**c**) Overlapping structure of the 5 predicted aptamer models (refer to Table 1). (**d**) Top binding pose for binding site 1 (Aptamer3_1). (**e**) Top binding pose for binding site 2 (Aptamer5_1). (**f**) Top binding pose for binding site 3 (Aptamer1_1).

Figure 2a–b shows the secondary and tertiary structure of the modeled aptamer. Modeled aptamer adopts hairpin configuration (Fig. 2c) containing a stem/helix (7 base pairs, dG4 → dG10 and dC29 → dC35), a hairpin loop (18 nucleotides, dT11 → dT28), and an external loop (13 nucleotides, dT36 → dA48), with a net free-energy (ΔG) was calculated to be − 11.50. Tertiary structure prediction by the 3dRNA v2.0 web server proposed 5 possible aptamer structures (Fig. 2c). The RMSDs of the aptamer backbones were calculated by setting Aptamer1 as the reference structure to assess the structural similarities between the generated aptamer models (Table 1). RMSD values improved when the region outside the hairpin loop and stem were excluded from RMSD calculation (apart from Aptamer2), which suggests that apart from models 2 and 3 (RMSD values > 3), the core structures between the modelled aptamer are remarkably similar to each other.

**Table 1:** Predicted aptamer structural comparison using RMSD value (APT1 as reference model).

Model	RMSD (Å)			
	Whole aptamer		Stem and loop only	
	Backbone	Bases	Backbone	Bases
Aptamer1 (red)	0	0	0	0
Aptamer2 (yellow)	4.6	4.3	5.6	5.2
Aptamer3 (green)	9.9	9.3	3,4	2.8
Aptamer4 (blue)	17.1	16.3	2.8	2.7
Aptamer5 (magenta)	6.8	6.3	1.8	1.1

Previous studies revealed that AFB1 may intercalates with DNA via adduct/covalent bond formation through its derivative AFB1-8,9-epoxide^71^. However, it has also been shown that non-derivatized AFB1 can also intercalate with DNA by a process which was primarily driven by van der Waals force and hydrogen bonding^72^. While this docking study cannot show adduct formation between the dG and AFB1, it may predict the approximate binding location of AFB1 within the immobilized aptamer structure. A molecular docking study was conducted to predict the binding site and pose of AFB1 in the modeled aptamer. The result indicates that the aptamer contains multiple AFB1 binding sites at 5′-GGG-3′ (binding site 1), 5′-GCA-3’ (binding site 2), and 5′-ACG-3′ (binding site 3) (Table 2). All binding sites were located at the stem of the aptamer. The overall result shows that most binding poses were located within binding site 3 (48%), followed by binding site 2 (32%) and binding site 1 (20%). Top binding poses (BP) from each aptamer model also reflected the overall result, with AFB1 bound to Aptamer1, Aptamer2, and Aptamer3 at binding site 3, Aptamer5 at binding site 2, and Aptamer4 at binding site 1 (Table 2). Figure 2d–f shows the top binding poses from each binding site (Green dG, Red dA, yellow dC).

**Table 2:** Overall docking results of Aptamer1-5 with AFB1.

Aptamer model	Binding site, binding affinity, and no. of intermolecular hydrogen bonding									
	BP1	BP2	BP3	BP4	BP5	BP6	BP7	BP8	BP9	BP10
Aptamer1	3, − 8.2, 1	2, − 7.5, 2	3, − 7.4, 1	2, − 7.2, 2	1, − 7.2, 1	3, − 7.2, 0	2, − 7.1, 0	3, − 7.1, 2	2, − 7.0, 1	1, − 7.0, 1
Aptamer2	3, − 8.1, 2	3, − 7.6, 2	1, − 7.2, 2	3, − 7.2, 0	3, − 6.9, 1	1, − 6.8, 0	3, − 6.8, 2	2, − 6.7, 0	2, − 6.7, 1	1, − 6.6, 1
Aptamer3	1, − 8.6, 2	1, − 8.3, 1	2, − 8.2, 1	2, − 8.2, 1	1, − 7.9, 2	2, − 7.9, 1	2, − 7.6, 1	3, − 7.5, 0	2, − 7.3, 1	3, − 7.3, 1
Aptamer4	3, − 7.4, 1	3, − 7.3, 2	3, − 7.2, 2	3, − 7.2, 2	1, − 7.2, 2	3, − 7.2, 1	3, − 7.1, 2	3, − 7.1, 2	3, − 7.0, 1	3, − 6.9, 0
Aptamer5	2, − 7.4, 2	2, − 7.3, 1	2, − 7.3, 1	3, − 7.2, 1	2, − 7.1, 0	1, − 6.9, 1	3, − 6.8, 1	3, − 6.8, 1	2, − 6.7, 1	3, − 6.7, 1

### Molecular dynamic analysis between free 50-mer aptamer and AFB1-bound 50-mer aptamer

**Figure 3:**
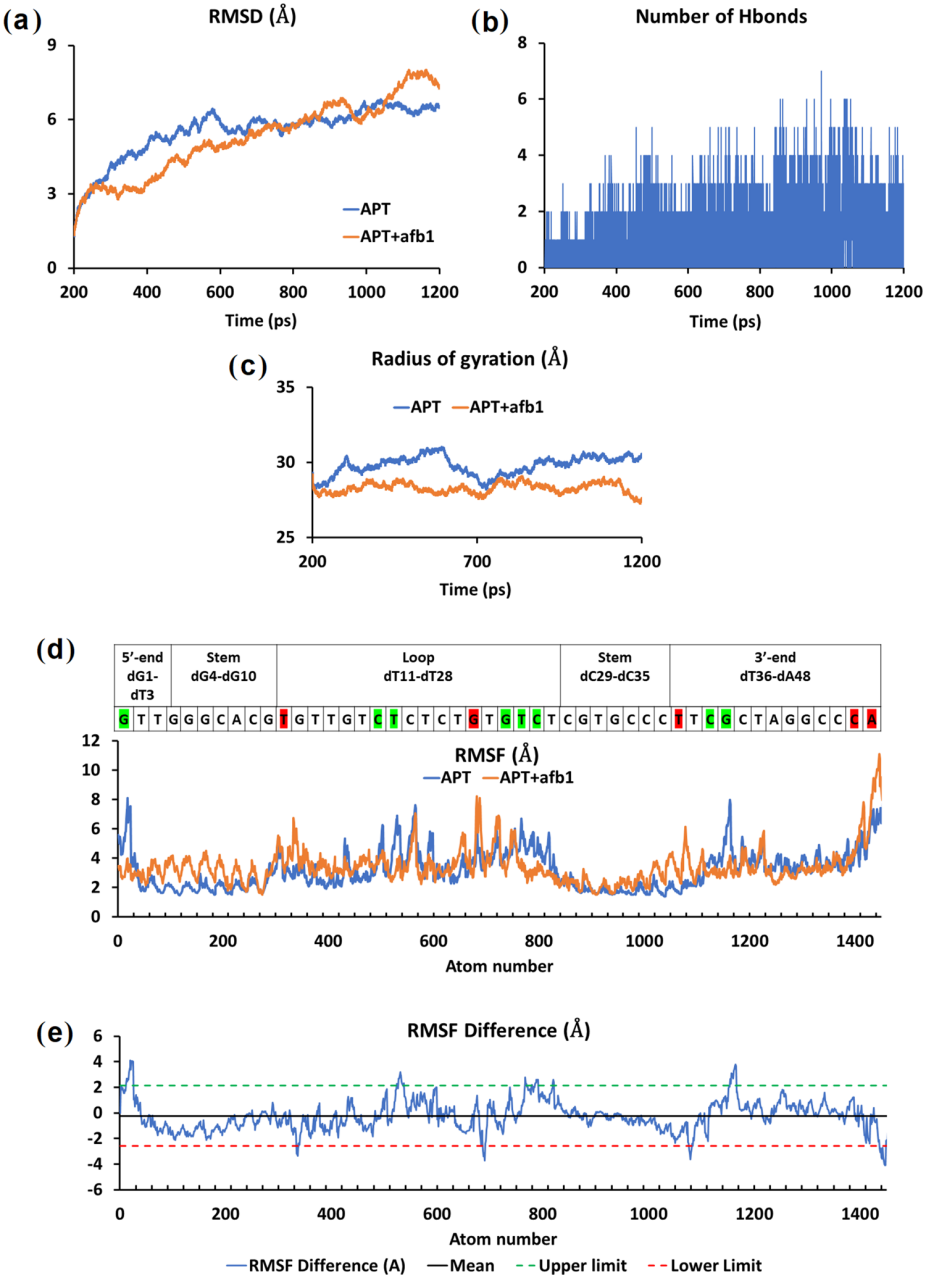
Molecular dynamic analysis. (**a**) RMSD of aptamers only (APT, blue) and aptamer-AFB1 complex (APT + afb1, orange). (**b**) Number of hydrogen bonds formed between aptamer and AFB1 throughout the simulation. (**c**) Radius of gyration of aptamer only (APT, blue) and aptamer-AFB1 complex (APT + afb1, orange). (**d**) RMSF values of aptamer (APT, blue) and aptamer-AFB1 complex (APT, afb1, orange). Nucleotides with root mean square fluctuation (RMSF) changes beyond two standard deviations from the mean were identified and color-coded: green for increased stability (+ 2.12 Å) and red for increased flexibility (− 2.60 Å). (**e**) A graph of RMSF difference between aptamer only vs. aptamer with AFB1.

In this mini molecular dynamic simulation (1 ns simulation time), the root mean square deviation (RMSD), root mean square fluctuation (RMSF), number of hydrogen bonds, radius of gyration (Rg), and Solvent Accessible Surface Area (SASA) were also analyzed to assess the properties of aptamer-AFB1 complex. The RMSD is calculated to determine the degree of free and ligand-bound aptamer deviation for each frame against a reference molecule throughout the simulation. The average RMSD for the aptamer-AFB1 complex and free aptamer throughout the simulation were 4.62 Å ± 2.00 and 4.84 ± 1.82 Å, respectively. The RMSD values, unchanged, indicate that AFB1 binding does not affect the stability of the overall aptamer structure (Fig. 3a). RMSF values were used to further evaluate the stability of the free and AFB1-bound aptamer at individual residue or nucleotide, with lower RMSF value usually corresponds to a more rigid biomolecule structure. The average RMSF values for free and AFB1-bound aptamer were 3.20 ± 1.32 and 3.44 ± 1.24 Å, respectively (Fig. 3d).

Figure 3e shows a closer look at the individual nucleotide RMSF values. The cut-off values for RMSF difference, which sufficiently represent significant nucleotide positional shift, were determined following a method by Salsbury (2010)^73^. A graph of the RMSF difference between complex and aptamer was first plotted (Fig. 3e), and nucleotides exhibiting a change in RMSF either exceeded the mean plus 2 standard deviations (+ 2.12 Å, green; increased stability) or lower than the mean minus two standard deviations (− 2.60 Å, red; increased flexibility) were noted and color-coded in Fig. 3d. The result shows that none of the nucleotides at the predicted binding site (stem) exhibited notable change in structural flexibility. Next, the average number of H-bonds offers hints about the intensity of the interaction between the receptor and the ligand. Throughout the MD simulation, the number of both dynamic and stable H-bonds were formed between the aptamer (dT2, dT3, dG4, dG5, dC33, dC34, dC35, dT36, dT37, and dC38 with occupancies ranging from the 0.01 to 20.9%) and AFB1 varies between 1 and 7 (Fig. 3b). In addition to that, the complex also exhibited multiple dynamic H-bonds within the stipulated simulation run time (1 ns). Finally, the radius of gyration (Rg) analysis revealed that AFB1 binding improves the stability of the aptamer slightly (Fig. 3c). The average Rg values of the aptamer-AFB1 and aptamer only were 28.31 Å and 29.82 Å, respectively. AFB1 binding to aptamer decreases the complex Rg by about 5.1%.

### Selectivity and sensitivity profile of UV-based sensor via aptamer-functionalized conducting graphene oxide-chitosan

**Figure 4:**
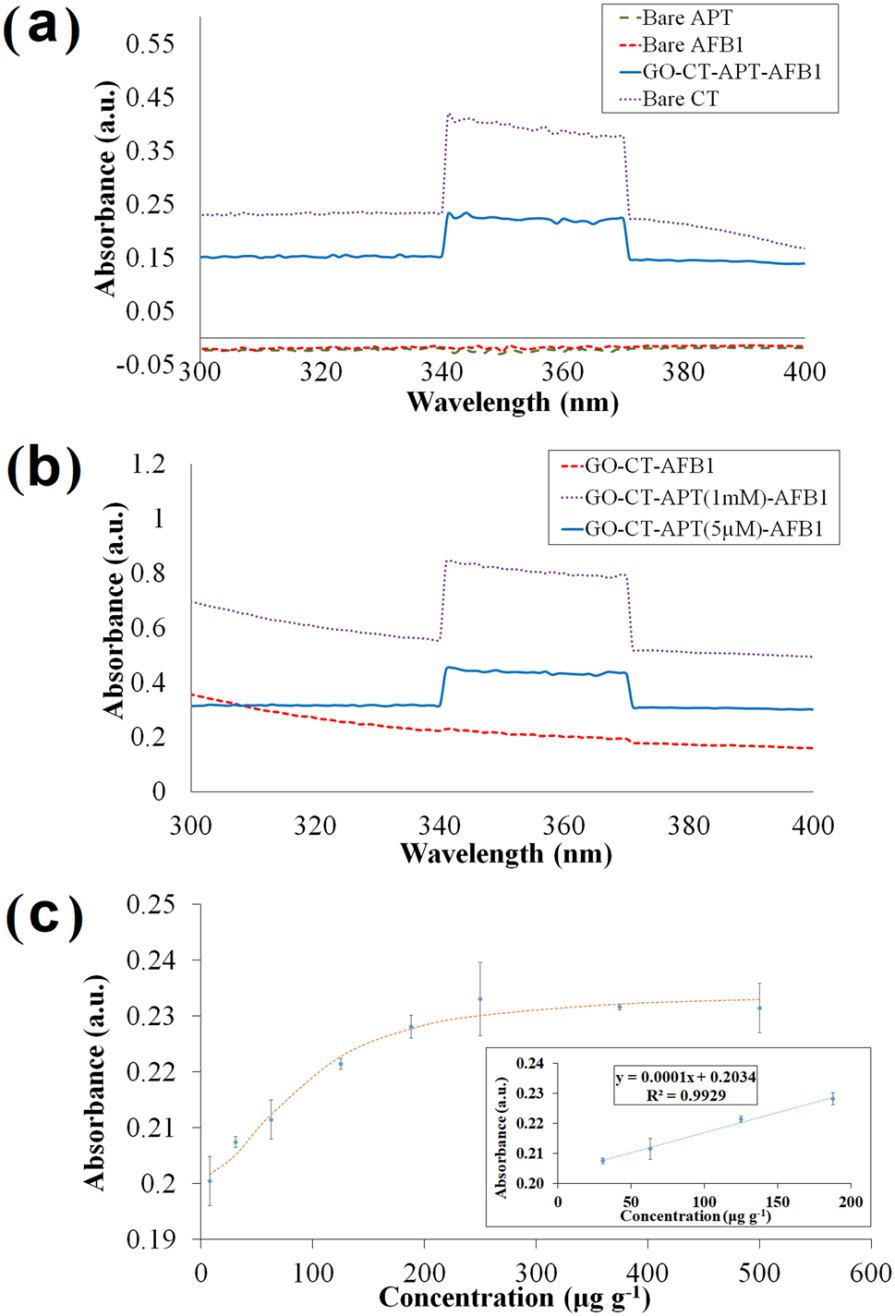
Selectivity and sensitivity profiles of UV-based sensor. (**a**) The selectivity spectral of bare materials and aptamer-functionalized conducting graphene oxide-chitosan with π–π stacked AFB1. (**b**) The UV spectral of AFB1 aptasensor in 1 mM versus 5 µM aptamer concentration. (**c**) A sigmoidal response curve of the optical aptasensor towards AFB1 concentration detection between 8 and 500 μg g^−1^. The inset shows a linear range profile from 71.8 to 188 µg g^−1^ with 23.7 μg g^−1^ detection limit AFB1.

As depicted in Fig. 4a, while bare aptamer and AFB1 were UV-inactive and showed no absorption peak, the aptasensor with conducting GO-CT undergoes electronic excitation when exposed to UV light, i.e., 100–400 nm during measurement, in contrast, no excitation signal when exposed to visible light. The broad peak between 340 and 372 nm was the characteristic peak for CT in CH_3_COOH solution, a peculiar and unique pattern ascribed to a set of NH_2_ groups present^74,75^. Upon AFB1 π–π stacking to the covalently bonded NH_2_ modified aptamer onto GO-CT, the aptasensor showed a discernible signal with an absorbance increment a.u. = 0.227 nm, which gave the highest reading, attributed to the functionalized conducting GO-CT (Fig. 4b). Molecular moieties bearing hetero atoms having non-bonding valence-shell electron pairs and pi-electron functions are likely to absorb light in from 200 to 800 nm, where only two electronic states of n → π* and π → π* undergo electronic excitation by the energies available within the ultraviolet–visible (UV–Vis) spectrum. When the aptasensor was exposed to light having an energy that matches a possible electronic transition within the molecule, i.e., stacked AFB1 moieties, some of the light energy will be absorbed as the electron is promoted to the n → π* and π → π* electronic states and thus signal changes by absorbance increment was yielded upon AFB1 stacking, the hyperchromic shift phenomenon. This apparent change clearly shows the role of GO cross-linked CT optical properties in highlighting the selectivity of immobilized aptamer capturing probe towards AFB1. The working wavelength at λ_max_ = 343 nm was the maximum UV measurement before and after the one-pot reaction.

According to the dynamic linear range of the sigmoidal curve illustrated in Fig. 4c, absorbance increased with more exposure to AFB1 in increasing concentrations, from 8 to 500 μg g^-1^. This trend was expected owing to more π-π interaction of stacked phenyl rings between complementary AFB1 and aptamer; thus, more π-electron clouds that were excitable by ultraviolet light only to the electronic states of π → π*/n → π* mixed transitions^76,77^. A plateau state was then reached between 250 and 500 μg g^-1^ AFB1, a saturation phase implying the unavailability of an immobilized aptamer probe to capture excess AFB1; therefore, constant values were given. The aptasensor was able to act at low concentrations where 62.5 μg g^-1^ AFB1 was employed in our subsequent studies, determined from the linear relationship profile ranging between 71.8 and 188.0 μg g^−1^, represented by the equation: y = 1.4028 × 10 − 4x + 0.20332 (R^2^ = 0.9889, LOD = 23.7 μg g^−1^, LOQ = 71.8 μg g^−1^).

### Optimal working conditions based on optical behavior of AFB1-sensing aptasensor

**Figure 5:**
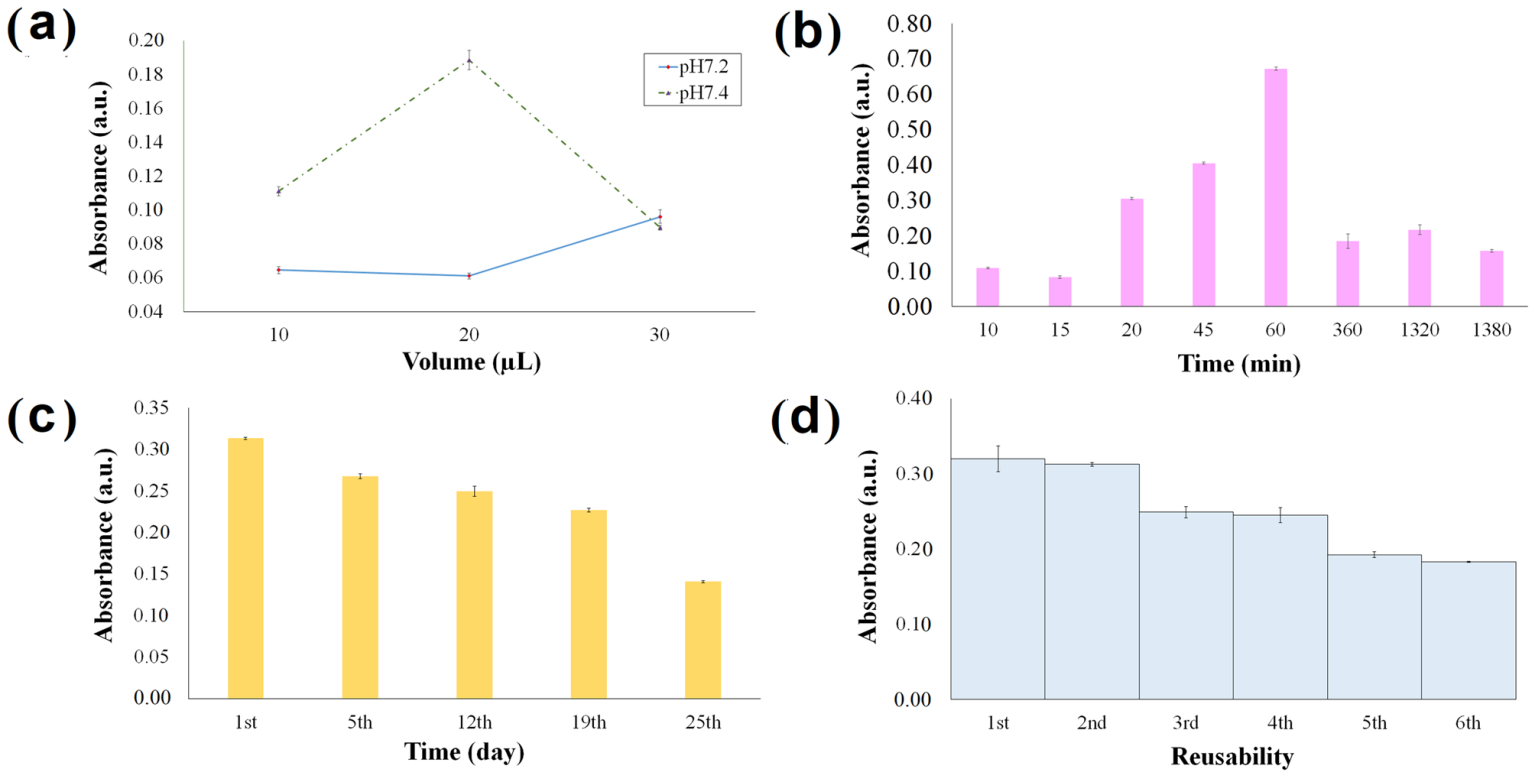
Optimal working conditions based on optical behaviour of AFB1-sensing aptasensor. (**a**) The response plots of EDC-NHS efficiency assessment with differing pH and loading volume. (**b**) The response time trend for the one-pot incubation between EDC, NHS, aptamer, and AFB1 on the GO-CT surface. (**c**) The solid-state aptasensor stability is maintained throughout 25 days of storage at room temperature. (**d**) The reversibility profile after consecutive loadings of non-modified aptamer on aptasensor was fabricated with a readily immobilized AFB1-targeted aptamer.

Based on Fig. 5a, using pH 7.4 Tris–HCl and 20 µL total volume gave the highest efficiency of EDC-NHS chemistry to facilitate aptamer immobilization onto GO-CT, enabling more π-based interaction from AFB1. The renowned “carbodiimide chemistry” used in bioconjugate and organic chemistry has been revealed to work in solutions and surfaces. EDC reacts with the remaining carboxyl (COOH) groups on the cross-linked nanocomposite via nucleophilic attack to form an unstable O-acyl isourea intermediate, which is NH_2_-reactive. After adding NHS, the NH_2_-reactive intermediate was converted into a semi-stable NH_2_-reactive NHS ester, leading to a more efficient conjugation to primary -NH_2_ at physiologic pH^78,79^. A two-step reaction upon added NHS increased the water solubility of an activated ester and the leaving group, hence more accessible substrates’ clean-up after activation^80,81^.

The incubation time needed for the one-pot reaction to go to completion is visualized in Fig. 5b. The aptasensor displayed an increasing response trend from 10 to 60 min before exhibiting a significant reduction, after which it continues up to 23 h. Before 20 min-incubation, the reaction rate was lower and more time was required for a higher rate of π-interaction, leading to unstable absorbance values. The prolonged incubation period, however, caused the loosely bound π-stacked AFB1 to dissociate from aptamer, and therefore, constant lessen readings were attained. A short incubation time of 20 min was chosen owing to the selectiveness and EDC-NHS intermediate reactivity, which significantly increased the binding rate between aptamer and AFB1.

Operational evaluation throughout the 25-day shelf-life study (Fig. 5c) revealed aptasensor stability for the first 19th days of storage at surrounding temperature, after which performance declined to 45% of their initial response. This suggested that covalent bonds formed not only between COOH of GO-CT and NH_2_ of aptamer, but also with NH_2_ of CT has rendered us long-term aptasensor stability with RSD reaches < 0.01. Figure 5d visualized the reversibility of aptasensor in detecting 62.5 μg g^−1^ AFB1 by regeneration using 50 μM n- aptamer within 20 min-reaction. Unsurprisingly, our aptasensor can be recycled up to four times (%RSD =  < 0.02) since aptamer can be denatured and refolded into a functional conformation for ‘n’ number of times^82^. By dispensing a higher concentration of non-modified aptamer, the AFB1- aptamer experienced a change in its configuration, causing AFB1 to dissociate from the aptamer probe. Continuous non-modified aptamer loading consequently led to the increasing number of AFB1 dissociations to form a newly π–π bound structure with non-modified aptamer giving 5^th^ and 6th regeneration signal lessens to about 40–43%.

### Validation of FEP-based aptasensor with standard HPLC–PDA technique

Spiked AFB1 in raw brown sugar, processed peanut, and long-grain rice samples were detected, and the results were comparable to High-Performance Liquid Chromatography-Photodiode Array (HPLC–PDA). The method’s limit of detection (LOD) was at 12.5 μg g^−1^, and the linear range for AFB1 standard calibration curve ranged from 37.8 to 500 μg g^−1^ at 362 nm. The relationship between the analyte concentrations and their UV absorbances is represented by the equation y = 3399x − 29,154 with an R^2^ value of 0.9988. Brown sugar, or non-centrifugal/cane brown sugar, is a raw, unrefined form of sugar produced by dehydrating sugarcane juice without centrifugation, which can be stored for 1–2 years. It is used as table sugar and a snack or as raw material for producing beverage, bakery, and confectionery products in Japan^83^. Peanuts are one of the essential nuts in the world, with the total production in 2019 equalling 48.8 million tons, which can be consumed as either processed products or raw materials, such as peanut butter, peanut oil, and roasted peanut^84^. Rice is available in more than 5000 varieties, where the exquisite aroma, tenderness, and sweet taste of long-grain rice have been regarded as “popcorn,” owing mostly to its major composition of 2-acetyl-1-pyrroline compound^85,86^. Recovery and precision results were compared against three different samples, as well as intraday and interday repeatability, as shown in Table 3. HPLC–PDA precision analyses, respectively, showed 9.9% (n = 9) intra-day and 10.9% (n = 9) inter-day repeatability (RSD), whereas the recovery (%) level of AFB1 from the spiked extracts was between 95.7% ± 5.8 (n = 3) and 108.5% ± 16.1 (n = 3). Our aptasensor yielded each intra-day and inter-day repeatability of 4% (n = 6) and 9.6% (n = 10), and then the recovery percentage was from 86.9% ± 3.4 (n = 3) to 102.7% ± 0.4 (n = 3).

**Table 3:** Recovery and precision comparisons between the proposed AFB1 aptasensor and standard HPLC–PDA technique.

Validation	AFB1 detection	UV-based aptasensor	HPLC–PDA
Recovery (%)	Brown sugar	102.7 ± 0.4, n = 3	108.5 ± 16.1, n = 3
	Peanut	86.9 ± 3.4, n = 3	85.8 ± 4.3, n = 3
	Rice	98.5 ± 0.4, n = 3	95.7 ± 5.8, n = 3
Precision (RSD %)	Intraday	9.4, n = 6	9.9, n = 9
	Interday	9.6, n = 10	10.9, n = 9

### Performance comparison with other reported AFB1-targeted aptasensors

Supplementary Tables 1 and 2 encompassed pre-analytical, analytical, and post-analytical factors influencing overall scientific observations and results, between the newly developed aptasensor and previously reported aptasensors^87–93^. Supplementary Table 1 tabulated the influencing factors in terms of design basis, capturing probe, capturing probe strategy approach, label, immobilizer, immobilizer interaction with probe, fabrication simplicity, and device principle for on-site or forensic detection. On the other hand, Supplementary Table 2 assessed in detail the probe selectivity profile in the absence or presence of target, linear range (μg g^−1^), detection limit (μg g^−1^), incubation time (min), reusability and regeneration capability, shelf-life, recovery, selectivity in beverages containing complex and interfering ingredients, and suggested conformation and relative orientation between probe and targeted AFB1 for illustrating recognition principle in AFB1 diagnostic tools.

Amino-caproic acid (6-aminohexanoic acid) incorporation as the spacer promotes conformation and mobility also increases strength for aptamer adsorption on the reduced graphene nanosheets, in which 60-min incubation was required^87^, a relatively longer amide interaction duration between immobilizer and probe, than that offered by our 20-min on-site fabrication simplicity with a high surface area-to-volume ratio of layered nanomaterial sheet. In contrast with our proposed label-free approach, which renders linearity of 71.8–188 µg g^−1^ with 23.7 μg g^−1^ detection limit, the widely employed methylene blue (MB) redox probe placement on aptamer terminal can increase the sensitivity of target-binding attributed to efficient delocalization of π-electrons^88^. However, MB may pose long-term toxicity and undergo photodegradation reaction when exposed to UV light^94^. Apart from the time-consuming electrode preparation involving multiple incubations of materials, an aptamer structure was observed to exhibit a switchable signal upon horseradish peroxidase (HRP)-labelled aptamer and APT-AFB1 complex formation, owing to biocatalytic precipitation induced by HRP^89^. In light of the reusability and regeneration capability, our constructed aptasensor offers an alternative to catalytic detection, enabling up to four times aptamer regeneration on its optical conducting solid-state platform, using amide bond formation, π-electron delocalization, EDC-NHS activation chemistry, and intercalation utilizing hydrogen bonding between alternating non-modified aptamer, modified-aptamer with multiple ligand binding sites at its stem, and AFB1 incubated materials.

The fluorescence probes of APT-modified carbon dots (CDs) utilizing humic acid immobilizer as the blue fluorescence quencher of the CDs attained aptasensor stability for up to fourteen days, attributed to the immobilizer π-interaction with probe^90^. An even higher stability of nineteen days was achievable in this study owing to the stronger amide bond formed between the GO-CT matrix and capturing aptamer, as the π-binding affinity of AFB1 ligands towards the stem of the immobilized aptamer having multiple binding sites. Another fluorescently-labeled aptamer with 5-carboxy tetramethylrhodamine (TAMRA) dye produced a long 55-min assay yielding 90.4–104.1% of AFB1 sample recoveries upon TAMRA label adsorption onto metal–organic frameworks^91^. Meanwhile, we obtained an 86.9–102.7% facile recovery of AFB1 samples containing complex and interfering ingredients, using a one-pot away label-free 20-min detection basis by ultraviolet-excitable conducting GO-CT. A label-free fluorescent aptasensor based on quaternized tetraphenylethene salt required a longer 90-min incubation time compared to our label-free aptasensor AFB1 detection approach, which only needed 20 min to give a stable UV-excitable spectrum upon reaction completion^92^. A lateral flow strip biosensor requires an additional preparation of the control line, by combining the remaining aptamer with biotin-probe 2, giving a 10-min rapid AFB1 detection achievable using fluorescent cyanine (Cy5) dye-labelled aptamer^93^. As for this study, we applied unique UV-active spectrum fingerprint of aptasensor based on hydrophobic FEP film strip feasible for AFB1 on-site/forensic detection.

### Performance comparison with conventional methods and commercially available kits for AFB1 detection

AFB1 detection technology can be categorized into immunological, chromatographic, biosensor, or combined approaches. Immunological methods^95^ and chromatographic^96^ are usually applied to detect AFB1. While these techniques offer good accuracy and sensitivity, certain drawbacks make them challenging for on-site detection (Supplementary Table 3). For example, although the instrument read time in Enzyme-Linked Immunosorbent Assay (ELISA) can be as short as 2 min, the requirement for multiple incubation and washing steps can be time-consuming, taking 24 h for direct uncoated ELISA kits, 2.5 to 4 h for indirect coated ELISA kits, and 3 h for instant ELISA kits. Furthermore, several practical challenges hinder its application due to its reliance on antibody-based immunoassays. These include extended reaction times, prohibitive costs, lack of real-time detection, difficulties in producing quality and stable antibodies through animal immunization (which can take several months), and antibody self-decomposition during storage^97^.

In contrast, High-Performance Liquid Chromatography (HPLC) allows for individual toxin quantification. However, chromatography-based methods are complex and demanding, necessitate expensive equipment and trained professionals, and are not portable^98,99^. AFB1 is less fluorescent than aflatoxin B2 and G2 due to its rigid and conjugated structure. Common practices involve minor structural modifications of AFB1 to improve fluorescence detection. Techniques that involve the application of cyclodextrins, pyridinium hydrobromide perbromide, pre-column photochemical derivatization with trifluoroacetic acid (TFA), or post-column photochemical derivatization with iodine are employed. These modifications enhance the fluorescence signal by eliminating interfering substances from the matrix. However, this approach is more complex and may not be straightforward for on-site use, particularly by non-experts^100^. LCMS is capable of analyzing trace amounts of food residues. Nevertheless, it involves additional steps, such as 30 min of shaking and 5 min of centrifugation at 1650 g, along with the evaporation of eluate to dryness at 40 °C under gentle nitrogen stream treatment. Moreover, since it uses mass spectrometry, it is a destructive method that breaks down samples into non-recoverable components. Therefore, it may be less suitable for non-experts and could result in unsatisfactory results if handled incorrectly after each run^101^.

Supplementary Table 4 showcased pre-analytical, analytical, and post-analytical factors influencing overall scientific observations and results, between the newly developed aptasensor and several commercially available kits for AFB1 detection. This table highlighted several paramount details, which encompasses the kit brand, manufacturer, kit technique, application principle, sample type, detection limit (μg g^−1^), incubation mode, kit shelf-life upon storing with sample, recovery rate (%), extraction solution, and on-site operability in environmental settings. The colorimetric detection card by AFB1(Aflatoxin B1) Lateral Flow Assay Kit (Elabscience®, USA), as well as our newly proposed UV-active aptasensor based on fluorinated ethylene propylene film strip, allows facile naked-eye observation suitable for on-site natural environmental monitoring of pollutant. Even though detection limits are comparable with or even lower than those demonstrated by instrumental methods, differences exist between the commercial ELISA-based kits^102^ such as MaxSignal® Aflatoxin B1 ELISA Kit (PerkinElmer, USA), Aflatoxin B1 (AFB1) ELISA Kit (Abcam, USA), and AgraQuant® Aflatoxin B1 ELISA Test (Romer Labs, Austria).

A wide range of AFB1-containing unprocessed and processed beverages were able to be detected by MaxSignal® Aflatoxin B1 ELISA Kit, using competitive enzyme immunoassay. On the other hand, AFB1-contaminated cane brown sugar, processed peanut, and long-grain rice comprising of other complex and interfering solution components were selectively identified employing peculiar UV-active spectrum fingerprint observation that were facilitated by greater surface area of negatively charged GO-CT adsorbent sheet. Exhibited only one day shelf-life upon storing with sample at 2–8 °C, another competitive enzyme immunoassay kit, namely Aflatoxin B1 (AFB1) ELISA Kit can give a good AFB1 recoveries from 70 to 120%, either using buffer or ethanol extraction solvent. In regard to this, our approach rendered a more stable optical biosensor kit upon sample storage, attributed by the strong amide interactions within the inter-surface of GO-CT and immobilized aptamer, high reactivity of carbodiimide groups, and multiple AFB1 π-interactions on the stem of the same capturing probe, and hence less leaching of aptamer structure from the layers of negatively charged hybrid nanocomposites. AgraQuant® Aflatoxin B1 ELISA Test employed a single MeOH extraction that was applied to seven other AgraQuant® mycotoxin kits, which leads to a two-step incubation measure to avoid deviation of detection limits from the actual results due to unfiltered co-extract inferring components of feedstuff, feed, grain, and edible oil. Meanwhile, the dual system of MeOH:Tris–HCl buffer extraction solution used in our one-pot approach deter beforehand, different co-extract inferring compounds from different matrixes while providing a conducive solution for nucleic acid stability and mobility, enhancing the recovery rate of 86.9–102.7% AFB1 environmental pollutant.

## Material and methods

### Instrumentation

Characterization of GO, CT, GO-CT nanocomposite, and fabricated aptasensors involved Fourier Transform-Infrared Spectroscopy (FTIR) and FESEM. ATR-FTIR spectra were recorded using Perkin Elmer Spectrum 400 FT-IR within the wavenumber range of 4000–400 cm^−1^, while FESEM images were captured by Zeiss Merlin/Merlin Compact/Supra 55VP. The graphite, GO, CT, and GO-CT XRD patterns were detected within the angular range of 10 − 30 on a Bruker D8 Advanced X-ray Diffractometer. The UV–vis spectral and absorbance of aptasensors were measured on a double beam JASCO V-370 UV–visible Spectrophotometer with light passing through at a 1 nm fixed bandwidth, 1,000 nm per min scanning speed, and 1 cm path length of semi-micro cuvettes.

### Chemicals and reagents

Chemicals and reagents were acquired from Sangon Biotech (graphite), EAM (MeOH, methanol, 99.9%) Thermo Scientific (EDC, > 98%), R&M Chemical (HCl, hydrochloric acid, 37%; HNO_3_, nitric acid, 65%), Friendemann Schmidt (CH₃COOH, acetic acid, ≥ 99.7%), Chemiz (H_2_O_2_, hydrogen peroxide, 35%; KMnO_4_, potassium permanganate, 99%; H_2_SO_4_, sulfuric acid, 95–98%; KCl, potassium chloride, 99–100.5%), BBI (CT, ≥ 95%), Aldrich (NHS, 98%), EMD Millipore Corporation (Tris base, tris(hydroxymethyl)aminomethane, 99%), HmbG (NaCl, sodium chloride, ≥ 99.0%; NaH_2_PO_4_.H_2_O, sodium dihydrogen phosphate monohydrate, 98–102%), Sigma Aldrich (NaOH, sodium hydroxide, 98%), Bendosen (K_2_HPO_4_, di-potassium hydrogen phosphate anhydrous, ≥ 98%), and Merck (ACN, acetonitrile, ≥ 99.9%). Both 50-mer 5′-amino (standard C6) modified and unmodified aptamer of AFB1 were purchased from Integrated DNA Technologies (IDT) (Supplementary Table 5). The AFB1 aptamer was patented by Neoventures Biotechnology Inc. (Canada) (Patent: PCT/CA 2010/ 001,292)^103,104^. AFB1 was purchased from Cayman Chemical.

The modified aptamer that binds specifically to AFB1 has an additional NH_2_ functional group at the 5′-end of 50-mer base sequences, enabling attachment onto the GO-CT immobilization surface. Both modified and non-modified aptamers were initially re-suspended using pH 7.5 Tris–EDTA (TE) buffer and subsequently incubated at room temperature for 30 min. After centrifugation at 10,000 × g for 1 min, aliquots were kept at − 20 °C storage condition. The 5 μM stock solution of aptamer was prepared stepwise through dilution in pH 7.5 folding buffer consisting of 0.01 M phosphate buffer and 1 mM MgCl_2_, annealing at 90 °C for 5 min, and cooling at 25 °C for 15 min. For fabrication purposes, further dilution of aptamer was done using Tris–HCl buffer. As for AFB1, 3 mg g^−1^ stock solutions were obtained by mixing MeOH and phosphate-buffered saline (PBS, pH 7.4) sequentially in a 1:5 volume ratio. The same buffer was used to dilute aliquots of AFB1-containing mixtures. EDC and NHS solution (200 mM) were prepared by dissolving 19.2 mg of EDC mixed with 500 μL H_2_O and 21.7 mg NHS in 500 μL Tris–HCl activation/coupling buffer at 25 °C.

### Synthesis and characterization of graphene oxide

Graphene oxide (GO) synthesis was conducted based on previously reported protocols but with slight changes^53,105^. Crude graphite powder (2 g) was exfoliated with 20 mL CH₃COOH in a 50 mL centrifuge tube via 5 h-sonification at ambient conditions. Then, it was neutralized (pH 7) by adding distilled water and filtered via vacuum-assisted filtration, followed by overnight oven-drying at 40 °C. A mixture of H_2_SO_4_ and HNO_3_ (40 mL) in a 3:1 volume ratio was slowly added to the pre-treated graphite in a 100 mL conical flask before 1 h vigorous stirring in an ice bath. Then, the solution was continuously stirred for 3 days at 35 °C after carefully adding 6 g of KMnO_4_, followed by 100 mL of H_2_O. After that, it was cooled to room temperature, and then H_2_O (200 mL) and H_2_O_2_ (5 mL) were added before stirring for 1 h. The supernatant was discarded after standing overnight, leaving the GO precipitate, which was rinsed with 10% HCl once. The resulting GO nano-platelets were dark brown, obtained after pH neutralization through repetitive H_2_O soaking and final oven-drying at 50 °C. XRD (Supplementary Fig. 2), ATR-FTIR (Supplementary Fig. 3), and FESEM (Supplementary Fig. 4) confirmed the molecular structure to describe the physical and chemical properties of the as-synthesized GO.

### Preparation of cross-linked nanocomposite of graphene oxide and chitosan

For fabrication purposes, 1% CT and 0.2% GO suspensions (w/v) were mixed beforehand to form GO-CT nanocomposite^53,105^. CT suspension (1%) was acquired by stirring overnight 1 g of CT powder in 100 mL of 0.05 M CH₃COOH organic solvent at 25 °C. On the other hand, a 30-min sonication of 0.02 g GO in 10 mL H_2_O was performed. Mixing individual suspensions at a volume ratio 3:2 was followed by 1 h-sonication and stirring for 12 h at ambient temperature, producing a homogenous GO cross-linked CT nanocomposite.

### Molecular docking study

Following previous literature, aptamer structural modeling was performed^106^. Mfold^107^, at the UNAFold web server (http://www.unafold.org), was used to predict the secondary structure from the nucleotide sequence. The aptamer sequence was formatted after designating an appropriate name for it. Then, it was modelled using the following parameters: (1) Constraint information: no information; (2) DNA sequence: linear; (3) folding temperature: 37 °C; (4) Ionic conditions: 1.0 mM Mg^2+^; (5) Correction type: Oligomer. All other parameters were left at default values (apart from the e-mail and image size).

After that, the corresponding secondary structure of the aptamer model obtained using Mfold was used for tertiary structure prediction using the 3dRNA function at the 3dRNA v2.0 web server (http://biophy.hust.edu.cn/new/3dRNA)^108^. The nucleotide sequence and the respective dot-bracket notation (Vienna file), obtained previously, were used as input. Then, the parameters were set as follows: (1) Molecule type: DNA; (2) Procedure: Default; (3) Sequence: aptamer sequence’ (4) Predict Second Structure: No; 5) 2D structure: aptamer secondary structure in dot-bracket format (can be obtained from reading the Vienna file obtained when predicting the nucleotide sequence secondary structure using Mfold); (6) # of Predictions: 5. The Procedure Optimize, 5 predictions, 3dRNALib2 and Minimization were used as advanced options. The tertiary structures with the lowest score (better statistical model) were saved as a Protein Data Bank (PDB) file.

Docking aptamer with AFB1 was performed using AutoDockVina (ADV) on the Chimera 1.16 interface. In ADV, the equation *c* = *∑*_*i*<*j*_* ft*_*i*_*t*_*j*_*(r*_*ij*_*)* represents the total conformation-dependent part of the scoring function. For a given conformation (i.e., the spatial arrangement of atoms), the equation sums the interaction energies *ft*_*i*_*t*_*j*_*(r*_*ij*_*)* for all pairs of atoms *i* and *j*, where *i* < *j*^109^. Each interaction energy is a function of the types of the two atoms and their distance. Before docking, the APT receptor structures were prepared using Chimera 1.16 software (Tools > Surface/Binding Analysis > DockPrep) (Shapovalov and Dunbrack, 2011). Hydrogens were added to the molecule, followed by charge designation by AMBER ff14SB for standard residues and Gasteiger for other residues. Then, the aptamer charge was neutralized by adding Mg^2+^ ions. On the other hand, the AFB1 structure was loaded into the Chimera 1.16 software (File > Fetch by ID > enter PubChem ID). The loaded AFB1 structure is then prepared for docking by first minimizing the structure (Tools > Structure Editing > Minimize structure) using the following parameters: (1) Steepest descent steps 100,000; (2) Steepest descent step size (Å): 0.02; (3) Conjugate gradient steps: 1000; (4) Conjugate gradient step size (Å): 0.02; (5) Update interval: 10.

Then, hydrogens were added to the molecule, followed by charge designation. After that, molecular docking analysis was done (Tools > Surface/Binding Analysis > AutoDockVina) to obtain each binding pose’s binding affinities of afB1. All five aptamer models were docked with AFB1, with the number of binding modes set at 10 to obtain the most binding poses for binding site prediction.

### Molecular dynamic simulation

Molecular dynamic simulation was run on an Aspire A512-52 laptop with the following specification: Intel(R) Core(TM) i5-8265U CPU @ 1.60 to 1.80 GHz processor, 20.0 GB (19.8 GB usable) RAM, integrated Intel (R) UHD Graphics GPU 620 GPU, and 64-bit operating system, × 64-based processor. NAMD software^110,111^ was used to do molecular dynamic simulations on docked receptor-ligand complex with the best binding affinity score to understand the dynamics and the receptor-bound ligand interaction behavior. The CHARMM-GUI website (https://www.charmm-gui.org/) generated the ligand topology file and its parameters for the NAMD application^112^. The receptor and the merged receptor-ligand complex structure PSF files were generated using visual molecular dynamics (VMD)^113^. Both the psf and pdb files of the merged receptor-ligand complex are solvated using the TIP3P method with a minimum box padding of 10 using the plugin in the VMD software. A cut-off distance of 10 Å was employed to calculate short-range nonbonded interactions. Long-range electrostatic interactions were assessed using particle mesh Ewald (PME) method^114^. The temperature was set at 310 K using the NVE method. Each simulation’s time step was set at 2 fs. The scaling factor was set at 1, with a dielectric constant of 1.0. VMD software was used for visualization and data processing. The system was minimized for 100,000 (frame 1–2000) steps, followed by running for 500,000 steps.

The molecular dynamics data analysis was done via VMD software. After the simulation data (PSF and DCD files) were loaded (File > New Molecule), the RMSD of the aptamer backbone (Extension > Analysis > RMSD Trajectory Tool) and hydrogen bond between the aptamer and AFB1 (Extensions > Analysis > Hydrogen bonds) analyses were performed via VMD interface. Tk console (Extensions > Tk Console) was used to source the tcl files for the RMSF, SASA, and Radius of gyration analyses, available in the Supplementary Document.

### Selectivity and sensitivity of aptamer-functionalized conducting graphene oxide-chitosan

The stepwise fabrication of the optically active aptasensor was done at 25 °C. GO-CT suspension (50 μL) was pipetted onto an FEP film strip, allowing it to dry. Then, 10 μL of EDC, NHS, aptamer, and AFB1 were dispensed onto the GO-CT. After that, it was left for 22 h before all non-bonding materials could be washed away before UV–vis measurements, while washing was carried out using copious amounts of 1 M Tris–HCl pH 7.4, 10 mM PBS pH 7.4, and H_2_O. Linear response range response was assessed through the ability of 5 μM immobilized aptamer to detect AFB1 in different concentrations between 8 and 500 μg g^−1^.

### Determination of optimal working conditions label-free and one-pot aptasensor

The effect of succinimide-carbodiimide chemistry of EDC/NHS mediation in activating -COOH groups at modified GO surface was studied through combinations of buffer capacity at pH 7.2 and pH 7.4, also total loading volumes of 10 uL, 20 uL, and 30 uL using 5 uM aptamer and 63 μg g^−1^ AFB1. To evaluate the incubation period, the aptasensor was introduced with 5 μM aptamer and 63 μg g^−1^ AFB1, to be recorded at intervals between 10 min and 23 h, via optimized EDC-NHS conditions, i.e., 20 μL loading volume at pH7.4 Tri-HCl. The regeneration study was performed by alternate aptasensor incubation with aptamer versus non-modified aptamer for 20 min at 25 °C, where this protocol was repeated six times. Shelf-life evaluation was conducted within 25 days until a significant diminution in the absorbance signal was perceived, where aptasensors were kept at 25 °C before subsequent measurement. The absorbance and spectral measurements were conducted in triplicate and prepared with a similar protocol.

### Calibration curve of AFB1 using HPLC–PDA

AFB1 standard stock solution (500 μg g^−1^) was prepared, and the samples were filtered using a 0.45 μm PTFE membrane filter for HPLC–PDA analysis. After filtration, two-fold serial dilutions were carried out to prepare concentrations ranging from 15 to 500 μg g^−1^ for the calibration curve (Supplementary Fig. 4). The limit of detection (LOD) and limit of quantification (LOQ) values were determined using the equations based on the method reported by ICH where LOD = (3.3 × δ)/S, and LOQ = (10 × δ)/S, where δ is the standard deviation of the Y-intercept and S is the slope of the linear regression equations (peak height vs concentration) (Guideline, 2005). Precision and recovery data of UV absorbance were also determined using the same reference, where for precision, AFB1 standard aliquots at 500, 250 and 15 μg g^−1^ were labelled and injected on the same day (intraday precision), and other sets were injected the day after (Interday precision).

The recovery data was obtained by spiking brown sugar, peanut, and rice extracts with 62.5 μg g^−1^ of AFB1. AFB1 standards and samples were analyzed in triplicate using Waters HPLC instruments. The column used was a reverse phase C18 column (2.1 ID × 150 mm, 140 3.5 μm, Waters Atlantis) was used at ambient temperature. An isocratic solvent system of 50% aqueous methanol was used as the mobile phase with a flow rate of 0.2 mL min^−1^ over a 20-min run time. The samples were injected at a volume of 1 μL, and the UV detection was carried out at 362 nm.

### Recovery determination of AFB1 in complex sample matrix

The practicality of the developed aptasensor was carried out in a complex sample matrix using the standard addition method for the recovery determination of AFB1. Eventually, the results of our aptasensor were validated with a slightly modified HPLC–PDA technique. Unrefined brown sugar, processed peanuts, and long-grain rice were purchased from a local market to prepare AFB1-positive samples. 5 mg of each sample were extracted at room temperature with 1 mL MeOH:Tris–HCl buffer at a ratio of 1:5 v/v, filtered through a disposable syringe filter (0.45 μm), spiked with standard AFB1 at 63 μg g^−1^, and subjected to vortex for 1 min.

## Conclusion

Through aptasensor experimental investigations, validation using a standard HPLC–PDA method, along with pictorial conformation and orientation assistance via molecular docking and dynamics, our novel constructed solid-state aptasensor was proven facile via label-free and one-pot sensing strategy yielding a distinctive spectrum fingerprint when exposed to UV light. The high recognition of AFB1 offers a selective platform for probing the integrity of aptamer 50-mer long base sequences. We are the first to construct a novel UV-based aptasensor based on an FEP film strip for AFB1 detection. The selectivity and reactivity of EDC-NHS intermediate towards NH_2_-end of aptamer allow aptasensor to act within a relatively fast incubation time of 20-min, exhibited high stability of 19th days shelf-life, was recyclable for four repeated use, satisfactory recovery in complex and interfering solution environment of natural cane brown sugar (102.7% ± 0.4, n = 3), peanut (86.9% ± 3.4, n = 3), and rice (98.5% ± 0.4, n = 3) samples which are comparable to that of HPLC–PDA method, hence offering a promising diagnostic platform for on-site monitoring or forensic application in a court of law.

### Supplementary Information

Supplementary Information.

## Data Availability

All data generated or analyzed during this study are included in this published article.
