# Supplementary material for: On-site sensing for aflatoxicosis poisoning via ultraviolet excitable aptasensor based on fluorinated ethylene propylene strip: a promising forensic tool
Source: Sci Rep. 2024 Jul 29;14:17357. doi: 10.1038/s41598-024-68264-3 (PMC11286874; doi:10.1038/s41598-024-68264-3)
Supplement: Supplementary file 1 — Supplementary Information. [file 41598_2024_68264_MOESM1_ESM.docx]

**Supplementary Information**

On-site sensing for aflatoxicosis poisoning via ultraviolet excitable aptasensor based on fluorinated ethylene propylene strip: A promising forensic tool

**Supplementary Figure**

**Supplementary Figure 1-3**

**Physical and chemical properties of solid-state AFB1 aptasensor**

Graphite is a layered material consisting of stacked graphene layers. Weak Van der Waals forces hold the layers together, and the layers themselves are composed of hexagonal carbon atoms arranged in a honeycomb lattice^1^. The X-ray diffraction (XRD) pattern of graphite shows a sharp and intense peak at 2θ angle of 26.6° corresponding to the (002) lattice plane reflection. Several other peaks in the pattern can also be assigned to various lattice plane reflections of the hexagonal graphite lattice structure. On the other hand, graphene oxide (GO) is a modified form of graphene that typically displays a much broader and weaker peak at 2θ = 12.4°, given by the presence of oxygen-containing functional groups, such as hydroxyl (OH) and epoxide (C-O-C) groups^2^. The peak broadening indicates a reduced degree of order in the graphene structure compared to pristine graphite (Supplementary Fig. 1).

Meanwhile, it is possible to examine the crystal structure and phase of the chitosan (CT) sample by contrasting the 2θ values acquired from the XRD pattern with well-known reference patterns. Two different diffraction peaks can be seen for CT, which are 10.5° (weak diffraction) and 21.5° (strong diffraction). The strong diffraction peak corresponds to the mixture of (001) and (100) planes, indicating an amorphous structure and monoclinic system of CT^2-4^. Both peak positions are characteristic fingerprints of semi-crystalline chitosan^5^. The small peaks in the GO-CT sample correspond significantly for CT and GO diffraction regions, respectively, showing the successful combination of GO and CT moieties.

Attenuated Total Reflectance-Fourier Transform Infrared Spectroscopy (ATR-FTIR) is commonly used to analyze chemical bonds and functional groups that exist in a material (Supplementary Fig. 2). ATR-FTIR spectrum of the as-synthesized GO, exhibited absorption bands at v = 3400-3200 cm^-1^ (OH stretching of COOH and OH groups), 1709 and 1620 cm^-1^ (C=O stretching of COOH groups)^6-8^, 1396 cm^-1^ (C-O stretching of C-O-C and alkoxy groups)^8^, 1260 cm^-1^ (O-H bending of OH groups)^9^, 1039 cm^-1^ (C-O and C-C stretching of COOH and C-O-C groups), 870 cm^-1^ (C-H and C-C out-of-plane bending mode of aromatic rings)^10^. Meanwhile, the most prominent peaks of CT were as follows: (1) The peak of 3245 cm^-1^ corresponds to the O-H and N–H stretching, denoting the presence of OH groups which controls water solubility; (2) The C-H stretching of the aliphatic group was responsible for the absorption band at 2823 cm^-1^; (3) Amide I band spotted at 1635 cm^-1^ was assigned for C=O stretching, used to assess how much deacetylation occurs, that influences solubility and biocompatibility properties; (4) Amide II band at 1550 cm^-1^ was associated with the N-H bending and C-N stretching, offering details on the hydrogen-bonded structure of chitosan; and (5) Band at 1407 cm^-1^ indicates the N-H bending of the primary amine (NH_2_) groups which control its cationic characteristics. Other significant absorption bands observed were the anti-symmetrical stretching of the C-O-C bridge at 1152 cm^-1^, C-O stretching of a saccharide structure at 1055 cm^-1^, and O–H bending at 1026 cm^-111^.

For GO-CT nanocomposite, there were several characteristic peaks observed in the ATR-FTIR spectrum: (1) The peak of 3353 cm^-1^ denoted the O-H stretching of OH groups in CT and GO; (2) The peak of 2860 cm^-1^ was related to the C-H stretching of the aliphatic chain in CT; (3) In contrast to pure CT and GO, the GO-CT spectrum exhibited shifting of absorption peaks to 1664 cm^-1^ and 1599 cm^-1^ which corresponds to the absorbance of the glycosidic link, the stretching vibration from C=O of CONH amide, and the N-H bending of NH_2_^12^. The addition of aptamer and AFB1 onto GO-CT does not result in additional peaks, and peaks remained unchanged except for the intensity shifting and changes for existing peaks of 1645 cm^-1^ and 1569 cm^-1^, attributed to the newly formed CONH linkages when aptamer immobilized via NH_2_-terminal to the remaining COOH on GO-CT, aside from those linkages between COOH of GO and NH_2_ of CT^13,14^. Specific chemical interactions between the different functional attributes of the GO-CT composite and AFB1-aptamer could contribute to these changes, and Field Emission Scanning Electron Microscopy (FESEM) may prove helpful in characterizing this phenomenon.

FESEM is a powerful imaging technique used to obtain high-resolution images of the surface morphology and structure of materials and provide valuable information on the dispersion and surface structure of the composite. GO are typically flat, thin, transparent sheets or flakes with a wrinkled or crumpled surface morphology due to oxygen functional groups on the surface (Supplementary Fig. 3a)^15,16^. The CT matrix is observed as a network of fibres or interconnected structures, thus having a fibrous appearance and sponge-like structure with an appropriate degree of aggregation (Supplementary Fig. 3b)^17^. A layered or stacked structure due to the heterogeneous distribution of GO sheets throughout the chitosan matrix was observed (Supplementary Fig. 3c). The intense interaction between chitosan and GO increased stiffness, resulting in a more robust composite material than a pure chitosan film^18^. Under the same magnifications, the surface characteristics, roughness, agglomeration, and distribution throughout our aptasensor in sensing AFB1 by the immobilized APT were revealed (Supplementary Fig. 3d). Significant cluster-like patterns suggested that AFB1-APT is present on GO-CT surfaces, indicating that the components are observably associated. Though FESEM analysis provides useful visual information, its resolution might not be enough to determine the precise nature of the interactions taking place at the molecular level between the materials. Thus, to clarify the complex molecular interactions at work, more analytical methods like molecular docking and dynamic prediction studies are necessary.


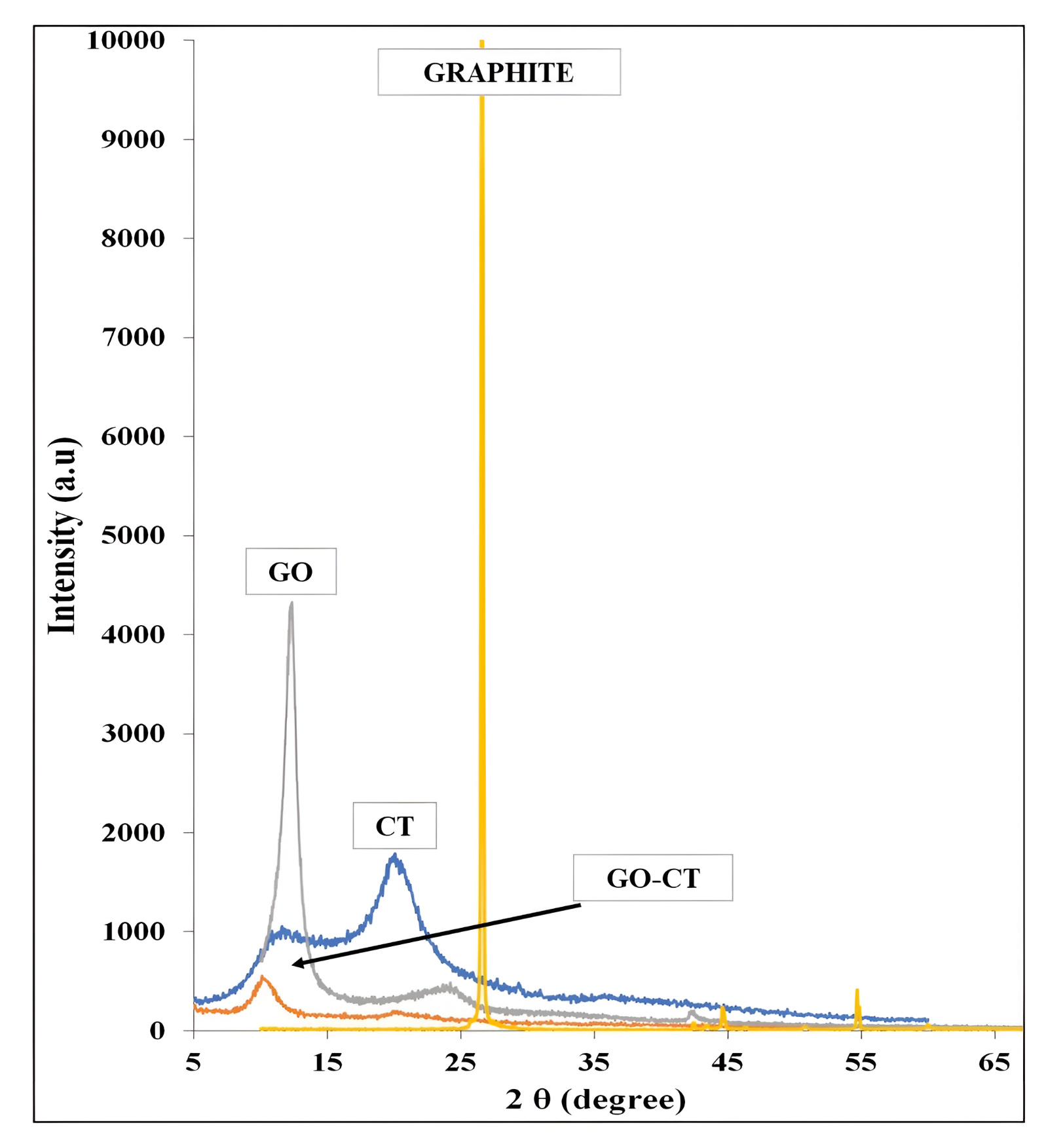


**Supplementary Figure 1. Comparison of X-ray diffraction (XRD) patterns of each layer** **generated by Bruker D8 XRD software using structures in ICSD.** The XRD pattern of graphite shows a sharp and intense peak at 2θ angle of 26.6° (yellow). XRD pattern for graphene oxide (GO) had a broader and weaker peak at 2θ =12.4° (grey). Two different peaks can be seen for chitosan (CT), which are 10.5° and 21.5° (blue). Small peaks in the GO and CT regions are proof of GO-CT moieties (orange).


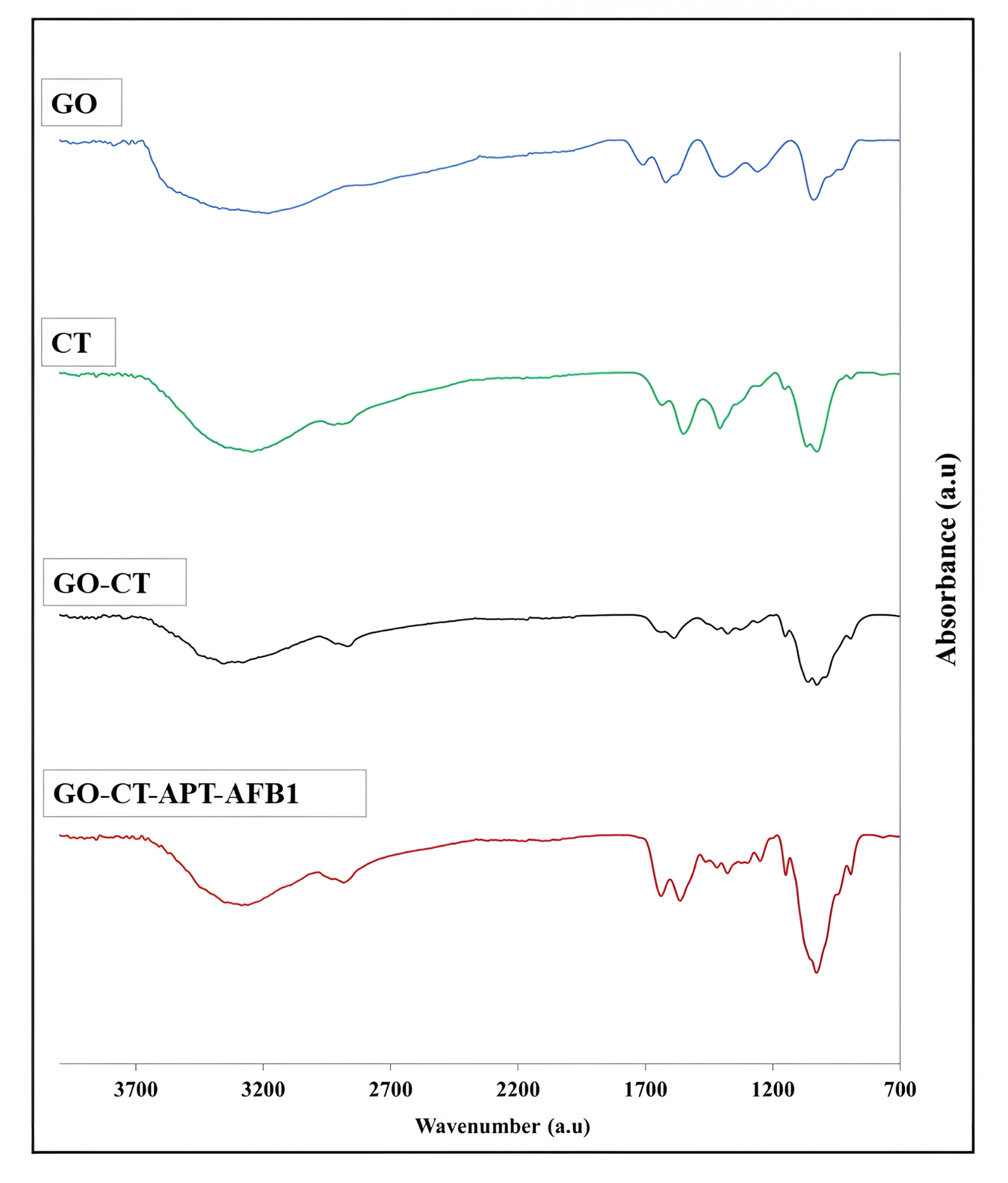


**Supplementary Figure 2. Attenuated Total Reflectance-Fourier Transform Infrared Spectroscopy (ATR-FTIR) spectra of graphene oxide (GO), chitosan (CT), GO cross-linked CT nanocomposite (GO-CT), and optical aptasensor detecting AFB1 on the GO-CT nanocomposite (GO-CT-PT-AFB1).** ATR-FTIRof GO ῡ (cm^−1^): 3400-3200 cm^-1^ (OH stretching of COOH and OH groups), 1709 and 1620 cm^-1^ (C=O stretching of COOH groups), 1396 cm-1 (C-O stretching of C-O-C and alkoxy groups), 1260 cm-1 (O-H bending of OH groups), 1039 cm-1 (C-O and C-C stretching of COOH and C-O-C groups), 870 cm-1 (C-H and C-C out-of-plane bending mode of aromatic rings). ATR-FTIR of CT ῡ (cm^−1^): 3245 cm^-1^ (O-H and N–H stretching), 2823 cm^-1^ (C-H stretching), 1635 cm^-1^ C=O stretching of Amide I, (4) 1550 cm^-1^ (N-H bending and C-N stretching of Amide II), and 1407 cm^-1^ (N-H bending of NH_2_). ATR-FTIR of GO-CT ῡ (cm^−1^): 3353 cm^-1^ (O-H stretching), 2860 cm^-1^ (C-H stretching), 1664 cm^-1^ and 1599 cm^-1^ (C=O stretching vibration of CONH amide, and the N-H bending of NH_2_). ATR-FTIR of GO-CT-PT-AFB1 ῡ (cm^−1^): 3353 cm^-1^ (O-H stretching), 2860 cm^-1^ (C-H stretching), 1645 cm^-1^ and 1569 cm^-1^ (intensity shifting and changes for existing peaks of CONH amide, and the N-H bending of NH_2_, attributed to the newly formed CONH linkages).


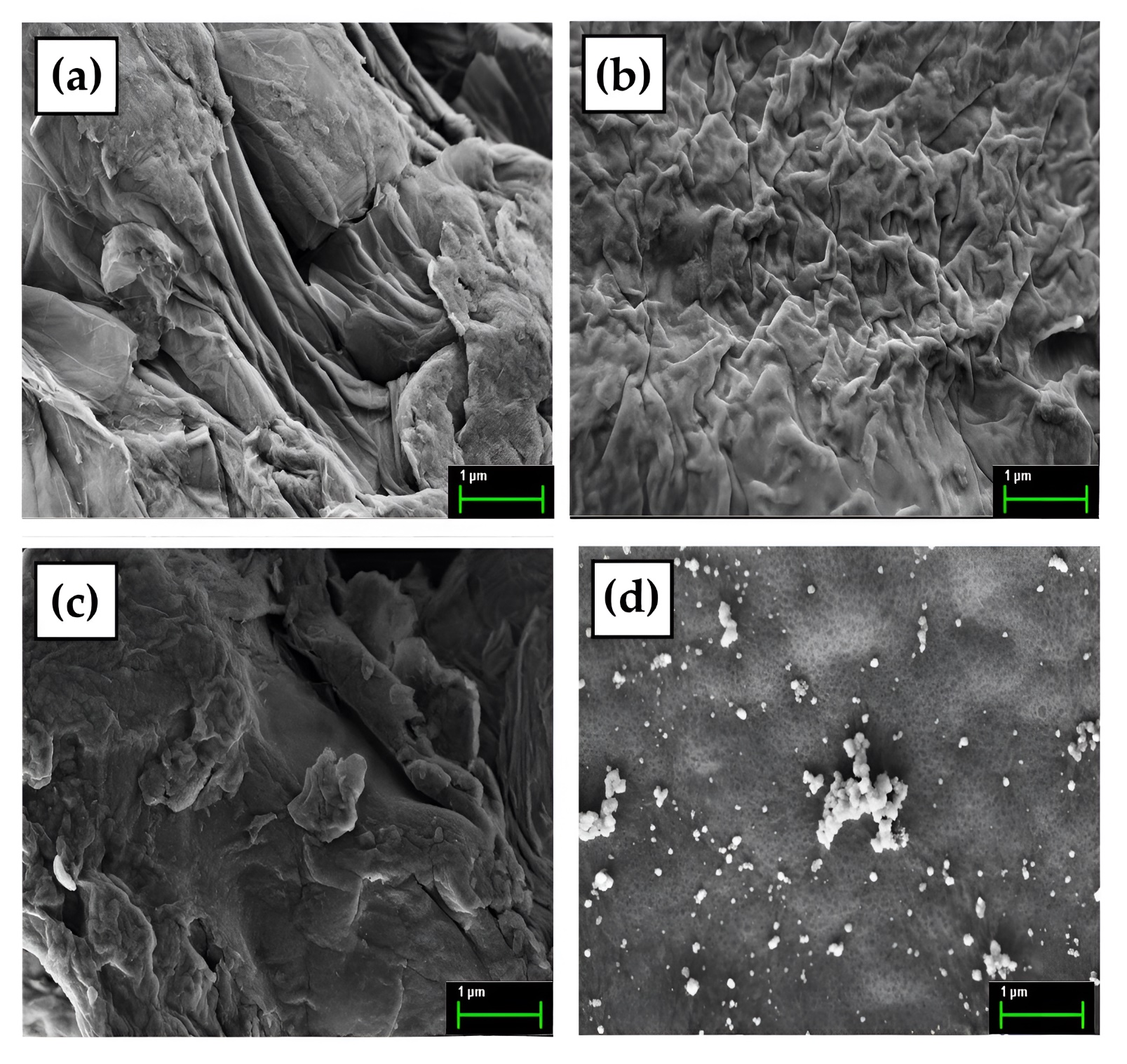


**Supplementary Figure 3. FESEM (Field Emission Scanning Electron Microscope) micrograph showing the morphological structure of respective layers.** For all FESEM examinations, samples were prepared by placing small pieces of sample films on the sample holder and analyzing images at 10 k X magnification. The numerical values represent the scale bars of the images (1µm). The graphene oxide (GO) image in (a) displayed surface wrinkling and folding, whereas (b) displayed the degree of chitosan (CT) aggregation with porous and sponge-like properties. The combined surface morphology of the GO-CT moieties, which included folding, wrinkling, porous, and sponge-like surfaces, was displayed in (c). Aflatoxin B1 (AFB1)-aptamer (APT) clump-cluster was observed on the surface layer of the composite GO-CT, as depicted in (d).

**Supplementary Figure 4**


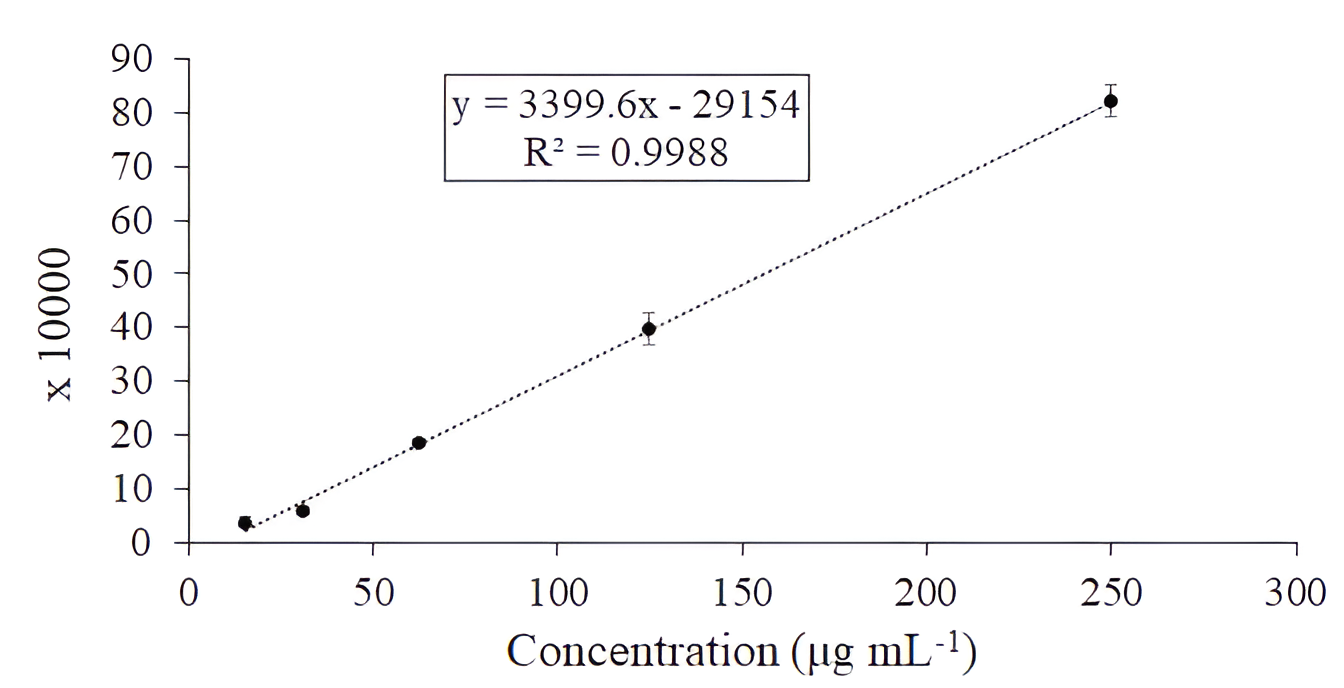


**Supplementary Figure 4. The calibration curve of AFB1 at 362 nm** **using High-Performance Liquid Chromatography-Photodiode Array (HPLC-PDA).** The limit of detection (LOD) and limit of quantification (LOQ) values were determined using the equations based on the method reported by ICH where LOD = (3.3 x δ)/S, and LOQ = (10 x δ)/S, where δ is the standard deviation of the Y-intercept and S is the slope of the linear regression equations (peak height vs concentration) (Guideline, 2005). Precision and recovery data of UV absorbance were also determined using the same reference, where for precision, AFB1 standard aliquots at 500, 250 and 15 μg g^-1^ were labelled and injected on the same day (intraday precision), and other sets were injected the day after (Interday precision).

**References**

1. Dhakate, S. R. et al. An approach to produce single and double layer graphene from re-exfoliation of expanded graphite. *Carbon* **49**, 1946–1954 (2011).
2. Chang, D. W. et al. Nitrogen-doped graphene nanoplatelets from simple solution edge-functionalization for n-type field-effect transistors. *J. Am. Chem. Soc*. **135**, 8981–8988 (2013).
3. Jia, Y. et al. A metal-organic framework/aptamer system as a fluorescent biosensor for determination of aflatoxin B1 in food samples. *Talanta* **219**, 121342 (2020).
4. Madian, N. G., El-Ashmanty, B. A., Abdel-Rahim, H. K. Improvement of chitosan films properties by blending with cellulose, honey and curcumin. *Polymers* **15**, 2587 (2023).
5. Dey, S. C. et al. Preparation, characterization and performance evaluation of chitosan as an adsorbent for remazol red. *Int. J. Latest Res. Eng. Technol*. **2**, 52–62 (2016).
6. Emiru, T. F. & Ayele, D. W. Controlled synthesis, characterization and reduction of graphene oxide: a convenient method for large scale production. *Egypt. J. Basic Appl. Sci.* **4**, 74–79 (2017).
7. Papaioannou, N. et al. Structure and solvents effects on the optical properties of sugar-derived carbon nanodots. *Sci. Rep*. **8**, 6559 (2018).
8. Galande, C. et al. Quasi-molecular fluorescence from graphene oxide. *Sci. Rep*. **1**, 85 (2011).
9. Zhang, T. Y. & Zhang, D. Aqueous colloids of graphene oxide nanosheets by exfoliation of graphite oxide without ultrasonication. *Bull. Mater. Sci*. **34**, 25–28 (2011).
10. Tanhaei, M., Mahjoub, A. R. & Safarifard, V. Ultrasonic-assisted synthesis and characterization of nanocomposites from azine-decorated metal-organic framework and graphene oxide layers, *Mater. Lett.* **227**, 318–321 (2018).
11. Fazial, F. F. & Tan, L. L. Phenylalanine-responsive fluorescent biosensor based on graphene oxide-chitosan nanocomposites catalytic film for non-destructive fish freshness grading. *Food Cont.* **125**, 107995 (2021).
12. Zuo, P. -P. et al. Fabrication of biocompatible and mechanically reinforced graphene oxide-chitosan nanocomposite films. *Chem. Cent. J*. **7**, 39 (2013).
13. Shao, L. et al. Graphene oxide cross-linked chitosan nanocomposite membrane. *Appl. Surf. Sci*. **280**, 989–992 (2013).
14. Valencia, A. M., Valencia, C. H., Zuluaga, F. & Grande-Tovar, C. D. Synthesis and fabrication of films including graphene oxide functionalized with chitosan for regenerative medicine applications. *Heliyon* **7**, e07058 (2021).
15. Arjun, N., Uma, K., Pan, G. T., Yang, T. C. K. & Sharmila, G. One-pot synthesis of covalently functionalized reduced graphene oxide–polyaniline nanocomposite for supercapacitor applications. *Clean Technol. Environ. Policy* **20**, 2025–2035 (2018).
16. Mutalib, M. A., Rahman, M. A., Othman, M. H. D., Ismail, A. F. & Jaafar, J. Chapter 9-scanning electron microscopy (SEM) and energy-dispersive X-ray (EDX) spectroscopy. *Membrane Characterization* ,161–179 (2017).
17. Khanmohammadi, M., Elmizadeh, H. & Ghasemi, K. Investigation of size and morphology of chitosan nanoparticles used in drug delivery system employing chemometric technique. *Iran. J. Pharm. Res.* **14**, 665–675 (2015).
18. Han, D., Yan, L., Chen, W. & Li, W. Preparation of chitosan/graphene oxide composite film with enhanced mechanical strength in the wet state. *Carbohydr. Polym*. **83**, 653–658 (2011).

**Supplementary Table**

**Supplementary Table 1. Construction design comparison between the proposed AFB1 aptasensor with other reported AFB1-targeted aptasensors.**

| Ref | Design basis | Capturing probe | Capture probe strategy approach | Label | Immobilizer | Immobilizer interaction with probe | Fabrication simplicity | Device principle for on-site or forensic detection |
| --- | --- | --- | --- | --- | --- | --- | --- | --- |
| This work | APT/EDC-NHS/GO-CT | 50-mer NH_2_-modified APT | Label-free | Not applicable | GO-CT | Amide formation | One-pot | UV |
| 87 | rGO-APT/ amino- caproic acid/EDC-NHS/ /GCE | 50 -mer C_6_-NH_2_- modified APT Amino- caproic acid(6-aminohexanoic acid) | Label-free | Not applicable | Modified GCE with amino- caproic acid as spacer | Amide formation | Multiple steps | DPV |
| 88 | Apt26-T18-MB | 26-mer SH-modified APT | Single-labelled | MB | Gold electrode  surface | Gold-sulfur chemistry | Multiple steps | SWV |
| 90 | HRP-APT/Co-WO_3_-AuNPs- ITO electrode | Aptamer structure switch of DNA_1_ coupled with HRP-induced  biocatalytic precipitation | Single-labelled | HRP | Co-WO_3_ nanorods-AuNPs  ITO electrode | Based on Au-S bands | Multiple steps | SPR |
| 91 | DNA-CDs/HAs | 59-mer amidogen modified APT | Single-labelled | DNA-CDs | HAs | π-interaction | Multiple steps | Fluorescence |
| 92 | TAMRA-APT/  UiO-66-NH_2_ | Aptamer labelled with TAMRA fluorophores | Single-labelled | TAMRA | UiO-66-NH_2_ based MOFs nanomaterial | Van der Waals  force | Multiple steps | Fluorescence |
| 93 | TPE-Z- APT/GO | 50-mer aptamer labelled with TPE-Z fluorophores induced AIEgens (do not require covalent functionalization with biomolecules) | Single-labelled | TPE-Z | GO | Electrostatic interactions | Multiple steps | Fluorescence |
| 94 | Cy5-APT/ streptavidin- biotinylated | Truncated Cy5-labelled aptamer and streptavidin- biotinylated  complementary strand | Dual-labelled | Cy5 and streptavidin-biotin | NC membrane | Anchoring of biotin-streptavidin conjugate on NC | Multiple steps | LFIA |

Note: APT = aptamer, GO-CT = graphene oxide crosslinked chitosan nanocomposite, NH_2_ = amine, rGO = reduced graphene oxide, EDC = N-(3-dimethylaminopropyl)-N′-ethyl-carbodiimide-hydrochloride, NHS = N-hydroxysuccinimide, GCE = glassy carbon electrode, SH = thiol, MB = methylene blue, HRP = horseradish peroxidase, Co-WO_3_ = cobalt-doped tungsten trioxide, AuNPs = nanorods/Au nanoparticles, ITO = modified indium tin oxide, DNA_1_ = short DNA strands, DNA-CDs = Aptamer-modified carbon dots, HAs = humic acid, TAMRA = 5-carboxytetramethylrhodamine dye, UiO-66-NH_2_ = amino-functionalised zirconium oxide lattice nodes, MOFs = metal-organic frameworks, AIEgens = aggregation-induced emission, TPE-Z = quaternised tetraphenylethene salt, DPV = differential pulse voltammetry, Cy5 = cyanine dye, NC = nitrocellulose membrane, UV = ultraviolet, SWV = square wave voltammetry, SPR = surface plasmon resonance, LFIA = lateral flow immunoassay

**Supplementary Table 2. Analytical performance comparison between the proposed AFB1 aptasensor with other reported AFB1-targeted aptasensors.**

| Ref | Probe selectivity profile in the absence or presence of target | Linear range (μg g^-1^) | Detection limit  (μg g^-1^) | Incubation time with target (min) | Reusability and regeneration capability | Shelf-life (day) | Recovery (%) | Selectivity in beverages containing complex and interfering ingredients | Suggested conformation and relative orientation between probe and targeted AFB1 for illustrating recognition principle |
| --- | --- | --- | --- | --- | --- | --- | --- | --- | --- |
| This work | Yes | 71.8 to 188 µg g^-1^ | 23.7 μg g^-1^ | 20 | Yes | 19 | 86.9–102.7 | Cane brown sugar, processed peanut, long-grain rice | Prediction using molecular docking and dynamic simulation |
| 87 | No data available | 1.6 × 10^-3^ µg g^-1^ – 1.3 µg g^-1^ | 2.2 × 10^-5^ µg g^-1^ | 60 | No data available | 7 | 94.6-104 | Pasteurized cow milk, human blood plasma | No data available |
| 88 | Yes | 2.5 × 10^-6^ μg g^-1^ to 7.8 × 10^-3^ μg g^-1^; 7.8 × 10^-3^ μg g^-1^ to 0.9 μg g^-1^ | 1.9 × 10^-6^ μg g^-1^ | 3 | Yes | 14 | Not applicable | White grape wine, milk, standard AFB1-  contaminated corn flour | No data available |
| 90 | Yes | 5 × 10^-9^ μg g^-1^ to 0.01 μg g^-1^ | 1 × 10^-9^ μg g^-1^ | 30 | No data available | No data available | 95–106 | Full-ester milk | No data available |
| 91 | Yes | 1 × 10^-4^ μg g^-1^ to 8 × 10^-4^ μg g^-1^ | 7 × 10^-5^ μg g^-1^ | 3 | No data available | No data available | 103.8–108.0 | Peanut oil | No data available |
| 92 | Yes | 1 × 10^-4^ μg g^-1^ to 8 × 10^-4^ μg g^-1^ | 3.5 × 10^-4^ μg g^-1^ | 55 | No data available | No data available | 90.4–104.1 | Milk, corn, rice | No data available |
| 93 | Yes | 0 – 3 × 10^-3^ μg g^-1^ | .2.5 × 10^-4^ μg g^-1^ | 90 | No data available | No data available | 91.4–95.1 | Milk, corn, rice | No data available |
| 94 | Yes | 2 × 10^-4^ μg g^-1^ to 0.02 μg g^-1^ | 1.6 × 10^-4^ μg g^-1^ | 10 | No data available | No data available | 93.3-112 | Peanuts, almonds, dried figs | No data available |

**Supplementary Table 3. Design and analytical performance comparison between our constructed UV-based aptasensor with** **several conventional techniques used for AFB1 detection.**

| Technique | Detection basis | Requirement | Solvent amount used | AFB1 derivatization in samples to enhance the detection | Relative Cost | Hands-on time per sample preparation | Read-out | Instrumentation read time | Portability | Require expertise |
| --- | --- | --- | --- | --- | --- | --- | --- | --- | --- | --- |
| Ultraviolet-based aptasensor | One-pot away detection by ultraviolet through employment of conducting GO-CT based on fluorinated ethylene propylene strip | - fluorinated ethylene propylene film strip inside ultraviolet cuvette - ultraviolet passing through the solid-state aptasensor | Lesser volume | None | Less expensive | 1 min | Ultraviolet | 2 min | Yes | No |
| High-Performance Liquid Chromatography - Ultraviolet / Fluorescence Detection | Utilize the pressure-driven flow of a mobile phase through a column packed with a stationary phase. | - C18 column packed with silica particles - Suitable ratio of solvent system mixture to achieve a good component separation | Larger volume | Not always | More Expensive | 1 min | Ultraviolet / Fluorescence Detection | Depends on the retention time | No | Yes |
| Liquid Chromatography coupled to Mass Spectrometry | Involves use of High-Performance Liquid Chromatography, wherein the individual components in a mixture are first separated followed by ionization and separation of the ions based on their mass/charge ratio. | - C18 column packed with silica particles - Suitable ratio of solvent system mixture to achieve a good component separation Direct Mass Spectrometry infusion for pure sample | Larger volume | None | More Expensive | 1 min | Mass Spectrometry | Depends on the retention time | No | Yes |
| Enzyme-Linked Immunosorbent Assay | Plate-based assay for detecting and quantifying soluble substances via core of immunochemistry is the specific interaction between immunoglobulin (Igs) and antigen (Ag) | - Typically performed in 96-well or 384-well polystyrene plates - Enzyme labels for signal detection generated via direct or secondary tag on the specific antibody (indirect) | Larger volume | Not always | More Expensive | 40 min – 1.5 h | Spectrophoto-meter, fluorometer, or luminometer | 2 min | No | Yes |

**Supplementary Table 4. Design and analytical performance comparison between our constructed UV-based aptasensor with several AFB1 commercial kits.**

| Commercial kit | Manufacturer | Kit technique | Application principle | Sample type | Detection limit  (μg g^-1^) | Incubation mode | Kit shelf-life upon storing with sample | Recovery rate (%) | Extraction solution | On-site operability in environmental settings |
| --- | --- | --- | --- | --- | --- | --- | --- | --- | --- | --- |
| This work | Not applicable | Ultraviolet-based aptasensor | Peculiar UV-active spectrum fingerprint | Cane brown sugar, processed peanut, long-grain rice | 23.7 | 20 min at 25 ℃ | 19 days stored at 25 ℃ | 86.9 – 102.7 | Methanol:Tris-hydrochloric acid buffer | Fluorinated ethylene propylene film strip |
| AFB1(Aflatoxin B1) Lateral Flow Assay Kit | Elabscience® (USA) | Lateral Flow Assay | Immunochromatography assay | Cereals, feed, oil | 0.005 – 0.02 | 8 – 10 min at 25 ℃ | No information available | No information available | Methanol; N-hexane | Colorimetric detection card |
| MaxSignal® Aflatoxin B1 ELISA Kit | PerkinElmer (USA) | Enzyme-Linked Immunosorbent Assay | Competitive enzyme immunoassay | Milk, milk powder, chicken, pork, beef, seed, feed, cereal, edible oils, pastries, peanut butter | 0.00004 | No information available | No information available | No information available | No information available | No |
| Aflatoxin B1 (AFB1) ELISA Kit | Abcam (USA) | Enzyme-Linked Immunosorbent Assay | Competitive enzyme immunoassay | Feedstuff, feed,  grain, edible oil. | 0.00015 – 0.00405 | 15 min at 20 – 25 °C | 1 day stored at 2-8 °C | 70 – 120 | Wash Buffer; Ethanol | No |
| AgraQuant® Aflatoxin B1 ELISA Test | Romer Labs (Austria) | Enzyme-Linked Immunosorbent Assay | Competitive enzyme immunoassay | Grains, nuts,  cereals, animal feeds | 0.002 | 5 – 20 min; No information available about temperature | No information available | No information available | Methanol | No |

**Supplementary Table 5. Base sequences of aptamers used in this study.**

| Types | 50-mer nucleotide sequences |
| --- | --- |
| Modified aptamer | *NH_2_*-5’-GTT GGG CAC GTG TTG TCT CTC TGT GTC TCG TGC CCT TCG CTA GGC CCA CA-3’ |
| Non-modified aptamer | 5’-GTT GGG CAC GTG TTG TCT CTC TGT GTC TCG TGC CCT TCG CTA GGC CCA CA-3’ |

**References (from main text)**

87. Beheshti-Marnani, A., Hatefi-Mehrjardi, A. & Es' haghi, Z. A sensitive biosensing method for detecting of ultra-trace amounts of AFB1 based on “Aptamer/reduced graphene oxide” nano-bio interaction. *Colloids Surf. B: Biointerfaces* **175**, 98-105 (2019).

1. Wang, C. & Zhao, Q. A reagentless electrochemical sensor for aflatoxin B1 with sensitive signal-on responses using aptamer with methylene blue label at specific internal thymine. *Biosens. Bioelectron.* **167**, 112478 (2020).
2. He, X. et al. Cobalt-doped tungsten trioxide nanorods decorated with Au nanoparticles for ultrasensitive photoelectrochemical detection of aflatoxin B1 based on aptamer structure switch. *Sens. Actuators B: Chem*. **332**, 129528 (2021).
3. Guo, M. et al. A simple aptamer-based fluorescent aflatoxin B1 sensor using humic acid as quencher. *Talanta* **205**, 120131 (2019).
4. Jia, Y. et al. A metal-organic framework/aptamer system as a fluorescent biosensor for determination of aflatoxin B1 in food samples. *Talanta* **219**, 121342 (2020).
5. Jia, Y. et al. A label-free fluorescent aptasensor for the detection of Aflatoxin B1 in food samples using AIEgens and graphene oxide. *Talanta* **198**, 71-77 (2019).
6. Zhao, Z. et al. A lateral flow strip based on a truncated aptamer-complementary strand for detection of type-B aflatoxins in nuts and dried figs. *Toxins* **12**, 136 (2020).

**Supplementary Codes for Molecular Dynamics Analysis**

**RMSF.tcl**

###################################################################################

set reference [atomselect top "nucleic" frame 1]

# the frame being compared

set compare [atomselect top "nucleic"]

set num_steps [molinfo top get numframes]

for {set frame 0} {$frame < $num_steps} {incr frame} {

# get the correct frame

$compare frame $frame

# compute the transformation

set trans_mat [measure fit $compare $reference]

# do the alignment

$compare move $trans_mat

}

set outfile [open RMSF.txt w]

set sel [atomselect top "nucleic"]

#puts $outfile "[measure rmsf $sel first 1 last 6000 step 1]"

set rmsf [measure rmsf $sel first 0 last 12000 step 1]

for {set i 0} {$i < [$sel num]} {incr i} {

puts $outfile "[expr {$i+1}] [lindex $rmsf $i]"

}

close $outfile

###################################################################################

**SASA.tcl**

###################################################################################

###############################################################

# sasa.tcl #

# DESCRIPTION: #

# This script is quick and easy to provide procedure #

# for computing the Solvent Accessible Surface Area (SASA) #

# of Protein and allows Users to select regions of protein. #

# #

# EXAMPLE USAGE: #

# source sasa.tcl #

# Selection: chain A and resid 1 #

# #

# AUTHORS: #

# Sajad Falsafi (sajad.falsafi@yahoo.com) #

# Zahra Karimi #

# 3 Sep 2011 #

###############################################################

puts -nonewline "\n \t \t Selection: "

gets stdin selmode

# selection

set sel [atomselect top "$selmode"]

set protein [atomselect top "protein"]

set n [molinfo top get numframes]

set output [open "SASA_$selmode.dat" w]

# sasa calculation loop

for {set i 0} {$i < $n} {incr i} {

molinfo top set frame $i

set sasa [measure sasa 1.4 $protein -restrict $sel]

puts "\t \t progress: $i/$n"

puts $output "$sasa"

}

puts "\t \t progress: $n/$n"

puts "Done."

puts "output file: SASA_$selmode.dat"

close $output

###################################################################################

**Radius of gyration (3 files)**

**1. center_of_mass.tcl**

###################################################################################

proc center_of_mass {selection} {

# some error checking

if {[$selection num] <= 0} {

error "center_of_mass: needs a selection with atoms"

}

# set the center of mass to 0

set com [veczero]

# set the total mass to 0

set mass 0

# [$selection get {x y z}] returns the coordinates {x y z}

# [$selection get {mass}] returns the masses

# so the following says "for each pair of {coordinates} and masses,

# do the computation ..."

foreach coord [$selection get {x y z}] m [$selection get mass] {

# sum of the masses

set mass [expr $mass + $m]

# sum up the product of mass and coordinate

set com [vecadd $com [vecscale $m $coord]]

}

# and scale by the inverse of the number of atoms

if {$mass == 0} {

error "center_of_mass: total mass is zero"

}

# The "1.0" can't be "1", since otherwise integer division is done

return [vecscale [expr 1.0/$mass] $com]

}

###################################################################################

**2. gyr_radius.tcl**

###################################################################################

proc gyr_radius {sel} {

# make sure this is a proper selection and has atoms

if {[$sel num] <= 0} {

error "gyr_radius: must have at least one atom in selection"

}

# gyration is sqrt( sum((r(i) - r(center_of_mass))^2) / N)

set com [center_of_mass $sel]

set sum 0

foreach coord [$sel get {x y z}] {

set sum [vecadd $sum [veclength2 [vecsub $coord $com]]]

}

return [expr sqrt($sum / ([$sel num] + 0.0))]

}

###################################################################################

**3. rog_loop_dcd.tcl**

###################################################################################

# load necessary tcl functions (Ref : http://www.ks.uiuc.edu/Research/vmd/vmd-1.7.1/ug/node182.html )

source gyr_radius.tcl

source center_of_mass.tcl

set outfile [open rg.dat w]

puts $outfile "i rad_of_gyr"

set nf [molinfo top get numframes]

set i 0

set prot [atomselect top "protein"]

while {$i < $nf} {

$prot frame $i

$prot update

set i [expr {$i + 1}]

set rog [gyr_radius $prot]

puts $outfile "$i $rog"

}

close $outfile

exit

###################################################################################
